# Dietary intakes, diet quality and physical activity levels from preconception to late pregnancy: Prospective assessment of changes and adherence to recommendations

**DOI:** 10.1177/17455057251341999

**Published:** 2025-06-24

**Authors:** Audrey St-Laurent, Anne-Sophie Plante, Stéphanie Harrison, Simone Lemieux, Julie Robitaille, Stephanie-May Ruchat, Anne-Sophie Morisset

**Affiliations:** 1 School of Nutrition, Laval University, QC, Canada; 2 Endocrinology and Nephrology Unit, CHU of Quebec-Laval University Research Center, QC, Canada; 3 Institute of Nutrition and Functional Foods, NUTRISS Research Center, Laval University, QC, Canada; 4 Department of Human Kinetics, Université du Québec à Trois-Rivières, QC, Canada

**Keywords:** nutrition, physical activity, preconception, pregnancy, prospective, energy, macronutrients, micronutrients, diet quality

## Abstract

**Background:**

Prospective nutritional and physical activity data are lacking throughout preconception and pregnancy.

**Objectives:**

To evaluate (1) intakes of energy, macronutrients and micronutrients, diet quality and physical activity levels in preconception and in each trimester of pregnancy and (2) adherence to recommendations.

**Design:**

Prospective study.

**Methods:**

Ninety individuals planning a pregnancy in the next year (Quebec, Canada) took part in four online assessments that occurred before conception and during each trimester of pregnancy (T1, T2 and T3). At each assessment, dietary intakes were derived from at least two web-based 24-h recalls, and supplements use was obtained from a web questionnaire. Diet quality was measured via the Healthy Eating Food Index 2019. Physical activity levels were evaluated with the International Physical Activity Questionnaire and the Pregnancy Physical Activity Questionnaire.

**Results:**

Preconceptionally, participants (30.5 ± 3.6 years) had a mean body mass index of 23.5 ± 3.4 kg/m^2^. Energy intakes (kcal/day) increased over time (preconception: 2172 ± 457; T1: 2284 ± 557; T2: 2382 ± 501; T3: 2434 ± 549; p < 0.0001), while Healthy Eating Food Index 2019 total score remained stable (p = 0.10). Although dietary fiber intake (g/day) increased from preconception to T3 (preconception: 23 ± 9; T1: 25 ± 9; T2: 26 ± 10; T3: 27 ± 9; p < 0.0001), more than 80% of individuals had daily dietary fiber intakes below 14 g/1000 kcal at each assessment. From preconception to T3, total intakes (foods + supplements) increased for iron, folate and vitamin D (p < 0.01), especially from preconception to T1. These intakes came mainly from dietary supplements and met recommendations for most individuals (>52%) at each assessment. Physical activity levels (METs – min/week) decreased from preconception to T3 (preconception: 1754 ± 1431; T1: 1518 ± 1124; T2: 1562 ± 1214; T3: 1258 ± 1218; p < 0.0001), whereas most individuals (64%–82%) complied with the physical activity recommendations at each assessment.

**Conclusion:**

Changes in dietary intakes and physical activity levels are observed from preconception to the end of pregnancy. The recommendations are met for most individuals, except for dietary fiber intakes. These results need to be confirmed in a larger, more heterogeneous sample.

## Background

Preconception (PC) can be described as the broad period during which an individual has the physiological ability to become pregnant^1,2^ or as a 2-year window preceding a pregnancy.^3^ Healthy eating habits and regular physical activity during PC and pregnancy are highly important for preventing and treating many periconceptional and prenatal complications, such as infertility, gestational diabetes mellitus, hypertensive disorders, insufficient or excessive gestational weight gain, preterm birth and large for gestational age neonates.^2,4–6^

It has been suggested that pregnancy is a window of opportunity for individuals to adopt healthier lifestyles, and individuals may be more motivated and prone to change their diet when becoming pregnant.^7^ Accordingly, a study revealed an increase in the frequency of fruit and vegetable consumption from before to early pregnancy (9–20 gestational weeks),^8^ and two other studies reported greater diet quality in pregnant individuals than in individuals of childbearing age or planning to conceive.^9,10^ However, to our knowledge, no study has prospectively evaluated diet quality from PC to pregnancy. A unique prospective study conducted by Cuco et al. among 80 Spanish individuals revealed small variations in the median energy intakes from PC to the 10th week of gestation (+97 kcal/day), from the 10th to the 26th week (−2 kcal/day) and from the 26th to the 38th week (−131 kcal/day), while the median intakes of macronutrients remained similar.^11^ These results are consistent with the suggestion that individuals planning a pregnancy increase their dietary intakes in anticipation of increased energy requirements later in pregnancy. In contrast, many studies have reported that individuals stabilize their dietary intakes and diet quality throughout gestation^12–14^ despite increased needs.^15,16^

As with diet, the PC period and each trimester present unique challenges and circumstances that can significantly influence physical activity levels, such as contraindications to prenatal exercise and the unfounded fear that physical activity could harm the safety of the fetus, per example.^17^ Cuco et al. demonstrated that the proportion of individuals who engaged in 30 min or more of physical activity at least 4 days/week linearly decreased from PC (18%) to late pregnancy (3%). These results are similar to the decrease in the volume (i.e. frequency, intensity and duration) of physical activity reported by other studies from PC to early pregnancy (<14 gestational weeks)^11,18^ and throughout gestation.^11,14,18–20^

To our knowledge, only the study conducted by Cuco et al. evaluated both dietary intakes and physical activity levels prospectively in PC and during each trimester among the same individuals.^11^ Further detailed evaluations of those parameters are needed to gain a comprehensive understanding of their changes and identify whether support is needed to achieve or maintain a healthy diet and regular physical activity during both periods. Thus, the aim of this study was to prospectively evaluate energy, macro- and specific micronutrient intakes, diet quality and physical activity levels from PC to late pregnancy and adherence to recommendations. We hypothesized that dietary intakes and diet quality would significantly increase from PC to early pregnancy and then stabilize until late pregnancy. We also anticipated that physical activity levels would linearly decrease from PC to late pregnancy. Finally, we hypothesized that adherence to dietary and physical activity recommendations is suboptimal during PC and pregnancy.

## Methods

### Study design and recruitment

**Figure 1.**
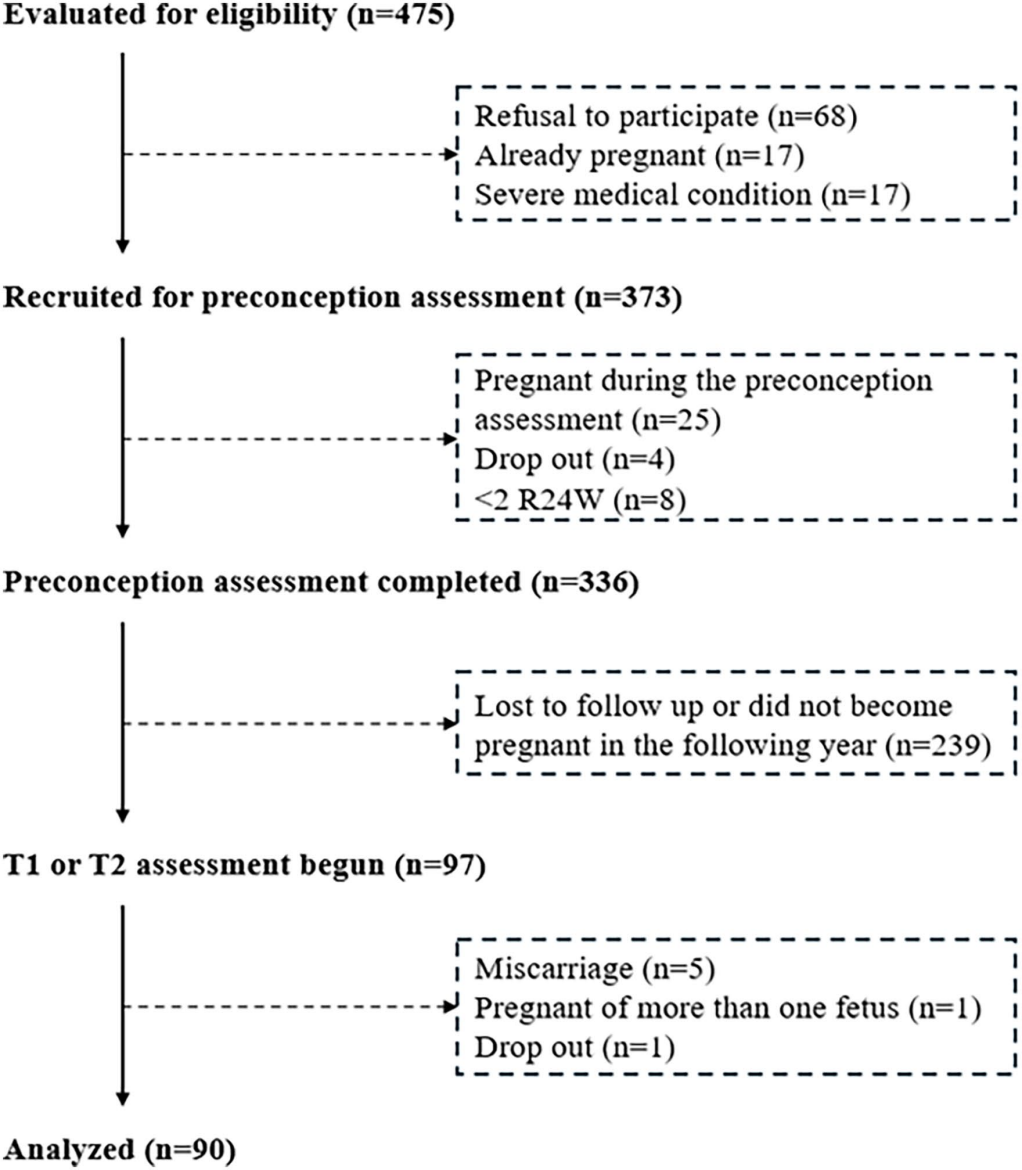
Flowchart.

The *ANGE-Contrôle-Enceinte* study is a prospective cohort study conducted in Quebec (Canada) from June 2017 to September 2023. Individuals planning to conceive within the next year were recruited through a multifaceted approach, including targeted social media advertising, email outreach to students and staff at Université Laval, and posters displayed in hospitals and research centers in the province of Quebec. The inclusion criteria were being ⩾18 years of age and planning a pregnancy in the following year. The exclusion criteria were being pregnant at the onset of the study and having a severe medical condition (e.g. type 1 or type 2 diabetes, renal or liver disease, inflammatory or autoimmune disorder, etc.). A total of 475 individuals expressed interest in the study. Informed consent was obtained from 373 individuals, 336 of whom completed the PC evaluation. Among them, 97 became pregnant in the following year and agreed to continue their participation during their pregnancy. Of those, 90 had nutritional and physical activity data available from at least three assessments (i.e. in PC and in at least two trimesters of pregnancy) and were included in the present study (Figure 1). The study was approved by the *Centre Hospitalier Universitaire de Québec – Université Laval* Research Center’s Ethics Committee (reference number: MP-20-2016-2866). This study is reported in accordance with the Strengthening the Reporting of Observational studies in Epidemiology – Nutritional Epidemiology guidelines.^21^ The completed checklist is available in Supplementary Materials 1.

Individuals completed a variety of web-based self-administered questionnaires within a 2-week period before pregnancy (PC: 5.0 ± 4.4 months before conception). All questionnaires used in this study are reported in Supplementary Materials 2. When the participants became pregnant, they were invited to continue with the project by completing questionnaires during each trimester (first trimester (T1): 9–12 gestational weeks; second trimester (T2): 22–25 gestational weeks; third trimester (T3): 33–36 gestational weeks). The questionnaires were hosted on a web-based platform named *Application fonctionnelle de nutrition sur internet* and are detailed below.

### Study population characteristics

In PC, participants completed a sociodemographic and a general health questionnaire to collect information on age, parity, annual household income, marital status, first language, ethnicity, education level, city of residence, weight, height and whether they were breastfeeding. Self-reported weight and height PC were used to calculate the prepregnancy body mass index. Gestational weights objectively measured by medical staff were obtained from medical records for 46.3% of the assessments conducted during pregnancy. Gestational age was calculated using the date of delivery estimated at the first prenatal echography.

### Dietary intake from foods

Multiple web-based 24-h recalls (R24W) were used to assess dietary intakes from foods. Three R24W recalls were sent over a 2-week period at each time point: in PC and at each trimester (12 R24W recalls throughout the study). An automated email was sent by the *Application fonctionnelle de nutrition sur internet* platform at midnight on randomly chosen days to prompt participants to complete each questionnaire. For each recall, the participants were asked to report every food and beverage they consumed on the previous day. All food entries within the database were encoded via the 2015 edition of the Canadian Nutrient File,^22^ enabling automated retrieval of nutritional data. Nutritional data were included if at least two R24W were completed per evaluation. The development and validation of the R24W among French-speaking pregnant or nonpregnant adult populations have been previously described.^23–26^

At each evaluation, intakes of energy, macro- and micronutrients from foods as well as the percentage of energy intake from carbohydrates, fats and proteins were obtained from R24W. The mean intakes were calculated from all available recalls. Each participant’s energy intake was compared to its respective estimated energy requirement for each assessment (PC, T1, T2 and T3). In 2023, the National Academies of Sciences Engineering and Medicine published updated equations to estimate energy needs. In the present study, the equations from 2005 were prioritized over those from 2023 for two main reasons. First, 92.3% of the study’s dietary measurements took place before the publication of the most recent dietary reference intakes for energy.^16^ Second, missing data on gestational weight for 46.3% of the measurements conducted during pregnancy made it impossible to calculate the estimated energy requirement using the 2023 formulas. Updated energy dietary reference intakes have been used only to identify the proportions of participants with energy intakes below, within or above their respective estimated energy requirements on the basis of one standard error of the predicted value, that is, ±241 in PC and ±302 during pregnancy.^16^

The intakes of carbohydrates, total fats and proteins as a percentage of total energy intake were compared to the acceptable macronutrient distribution range. Proportions of participants with intakes below, within or above the acceptable macronutrient distribution range were obtained from these comparisons. The daily estimated protein requirements were calculated using 0.66 g/kg of weight reported before pregnancy at PC and T1 (gestational week <20) and using 0.88 g/kg of weight reported before pregnancy at T2 and T3.^15^ The mean protein intake of the participants was compared to their estimated protein requirements. The intakes of dietary fiber of each participant were also compared to their respective needs, estimated to be 14 g/1000 kcal.^15^

### Micronutrient intakes from supplements

The participants completed a web-based self-administered questionnaire in PC and in each trimester to assess the use of vitamin and mineral supplements. For each supplement reported, participants had to specify the name, drug identification number, measurement unit, dosage and frequency of use per day. Up to 10 micronutrient supplements can be added. When needed, a research assistant entered additional information on the supplements reported by participants via the Health Canada Licensed Natural Health Product list^27^ as well as product labels from companies’ websites. The intakes of micronutrients from dietary supplements were derived via this additional information or the drug identification number when available. The participants were considered to have met the recommendations if they took a multivitamin of at least 400 µg of folic acid in PC or a multivitamin of at least 400 µg of folic acid and 16 mg of iron during pregnancy.^28,29^

### Total micronutrient intakes

Total micronutrient intakes were derived from the sum of the mean daily dietary intakes from foods and supplements. Total folate intake refers to the sum of the daily dietary folate intakes (natural folate from foods and folic acid from fortified foods) and the daily folic acid intakes from supplements. To calculate total folate, folic acid intakes (µg) were divided by 0.6 to be converted into folate (dietary folate equivalent (DFE)) as 1 DFE equals 0.6 of folic acid in µg.^15^ The total intake of folic acid refers to the sum of folic acid from fortified foods and supplements. The proportions of participants with micronutrient intakes below the estimated average requirement (EAR) and above the tolerable upper intake level (UL) were calculated when applicable.^15^

### Diet quality

In addition to documenting the quantity of nutrients consumed via R24W, diet quality was estimated via the Healthy Eating Food Index 2019 (HEFI-2019), which is the most recent index developed to measure adherence to the 2019 Canada’s Food Guide.^30^ The development and validation of this index have been described elsewhere.^30,31^ The HEFI-2019 total score (/80) is the sum of the scores of its 10 components: seven are “adequacy” components (vegetables and fruits (/20), whole-grain foods (/5), grain foods ratio (/5), protein foods (/5), plant-based protein foods (/5), beverages (/10) and the fatty acid ratio (/5)), and three are “moderation” components (saturated fats (/5), free sugars (/10) and sodium (/10)). The score of each HEFI-2019 component was calculated using the means of daily intakes from foods reported in all available R24W in PC and each trimester of pregnancy. Each HEFI-2019 component is expressed as a ratio of intakes in terms of densities or proportions to reflect the quality of food choices rather than the quantity of food consumed. Greater relative consumption of “adequacy” components and lower relative consumption of “moderation” components result in higher scores. A higher HEFI-2019 total score reflects better adherence to Canada’s Food Guide and thus better diet quality.

### Physical activity

Physical activity levels were assessed in PC using the short version of the International Physical Activity Questionnaire (IPAQ) in French. The IPAQ is a subjective tool validated in adults to assess different domains of physical activity (i.e. walking at work, for transportation, and for leisure, sport activities of moderate and vigorous intensities and sitting behaviors).^32^ The participants reported the frequency and duration of all walking and physical activities of moderate and vigorous intensities and their daily sitting time over the past week. According to the IPAQ scoring instructions, walking, moderate-intensity activities and vigorous-intensity activities were assigned values of 3.3, 4.0 and 8.0 METs, respectively. Physical activity levels during pregnancy were assessed via the French version of the Pregnancy Physical Activity Questionnaire (PPAQ).^33,34^ The PPAQ is a subjective tool used to assess prenatal physical activity in the past month.^35^ Predetermined activities (n = 34) are classified into six domains: household/caregiving (n = 9), occupational (n = 5), sports/exercise (n = 9), transportation (n = 3), work (n = 4) and inactivity (n = 4). In the sports/exercise domain, there were two open-ended questions allowing the participants to add any activities not previously listed. The participants reported the time spent per week on various predetermined activities. Each activity reported in the PPAQ is assigned an MET value according to the PPAQ scoring instructions and the compendium of physical activity.^35,36^ The activities were sedentary (<1.5 METs), light intensity (1.5–2.9 METs), moderate intensity (3.0–6.0 METs) or vigorous intensity (>6.0 METs). However, to harmonize the METs attributed to moderate- and vigorous-intensity activities across the IPAQ and PPAQ, the PPAQ MET values were changed to those of the IPAQ (3.3, 4 and 8) for the activities classified as walking, moderate intensity and vigorous intensity, respectively. This harmonization of MET values was necessary to ensure that changes in physical activity from PC to pregnancy were attributed to actual changes in behavior rather than discrepancies in the METs used for the same category of intensity in the IPAQ and PPAQ. For this study, total METs – min/week were obtained from the sum of METs – min/week devoted to all physical activities of moderate or vigorous intensity (walking, sports and leisure) across all physical activity domains. Physical activity levels in PC and at each trimester of pregnancy were also categorized into meeting the Canadian physical activity guidelines or not. The national recommendations suggest engaging in at least 150 min/week of moderate-to-vigorous physical activity in PC^37^ and at least 150 min/week of moderate physical activity during pregnancy.^38^

### Statistical analyzes

The study population characteristics are presented as the means and standard deviations for continuous variables and proportions (n, %) for categorical variables. To evaluate changes in dietary intakes, diet quality and physical activity levels (min/week and total METs – min/week) over time, repeated measures mixed models with random (participant number) and fixed (time: PC, T1, T2, T3) effects were used. If the residuals of the mixed models were not normally distributed, the value of the continuous outcome for each observation was transformed using a Box–Cox transformation. If the residuals of the mixed models were still not normally distributed after that transformation, rank regressions were computed with random (participant number) and fixed (time: PC, T1, T2, T3) effects. When an overall p < 0.05, multiple comparisons across time points were performed via a pairwise test. The p-values obtained from this test were adjusted via a Tukey–Kramer correction. Changes over time for categorical variables (i.e. the proportion of participants with dietary intakes below the EAR or above the UL and the proportion of participants meeting the Canadian physical activity guidelines) were evaluated using nominal logistic regression. When an overall p < 0.05, multiple comparisons across time points were performed via the McNemar–Bowker test. The p-values obtained from this test were multiplied by six (the total number of comparisons between time points) to adjust for the Bonferroni correction. Sensitivity analyzes were conducted to estimate the impact of breastfeeding in PC on changes in dietary intakes and physical activity levels from PC to late pregnancy, as well as adherence to recommendations. This was done by excluding women who were breastfeeding at PC and comparing the results to the full sample. A p < 0.05 was considered to indicate statistical significance. All the statistical analyzes were carried out via JMP^®^ Pro software version 16.2.0 (SAS Institute, Inc., Cary, NC, USA).

## Results

### Study population characteristics

Most participants were in their 30s, nulliparous, Caucasian and had a relatively high socioeconomic status (Table 1). The mean prepregnancy body mass index was 23.5 ± 3.4 kg/m^2^, and a majority of the participants were living with a partner in an urban area. In PC, 12.9% of the participants were breastfeeding, and participants became pregnant ~5 months after their PC assessment. The gestational age in weeks varied from 8.7 to 14.6 at T1, 21.4 to 24.0 at T2 and 32.1 to 35.4 at T3.

**Table 1.** Characteristics of the study sample in preconception (n = 90).

Variables	Mean ± SD or n (%)
Age (years)	30.5 ± 3.6
Nulliparous, yes	54 (60.0)
Body mass index (kg/m^2^)	23.5 ± 3.4
Underweight, <18.5	1 (1.1)
Normal weight, 18.5–24.9	64 (71.1)
Overweight, 25–29.9	21 (23.3)
Obese, ⩾30	4 (4.5)
Ethnicity	
Caucasian	87 (96.7)
Hispanic	3 (3.3)
Education level	
University, yes	73 (81.1)
Marital status	
Single	7 (7.8)
Divorced	2 (2.2)
Living with a partner	80 (88.9)
Prefer not to answer	1 (1.1)
Annual household income^a^	
<60,000$	24 (26.7)
60,000–79,999$	8 (8.9)
80,000–99,999$	19 (21.1)
⩾100,000$	37 (41.1)
Prefer not to answer	2 (2.2)
Living area	
Rural	17 (18.9)
Urban	48 (53.3)
Suburban	25 (27.8)
Breastfeeding^b^, yes	11 (12.9)
Time to conception following preconception assessment (months)	5.0 ± 4.4

CAD: Canadian dollar; SD: standard deviation.

a Income is presented in CAD.

b n = 85.

### Dietary intakes

At each assessment, most individuals completed three R24W (PC: 90%; T1: 87%; T2: 82%; T3: 80%, data not shown) while the remaining individuals completed two R24W.

#### Energy intakes

**Figure 2.**
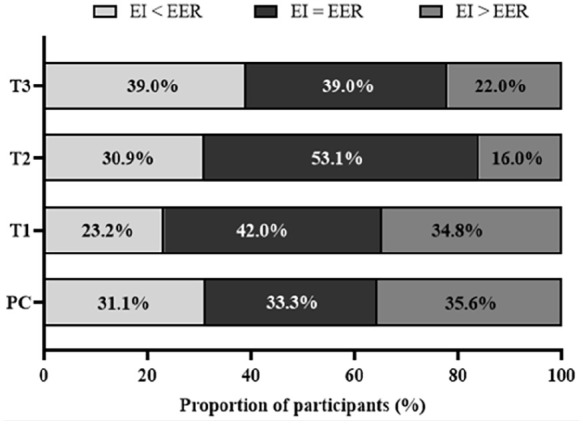
Proportion of participants below or above energy requirements in PC and in each trimester of pregnancy. EER: estimated energy requirements; EI: energy intakes; PC: preconception (n = 90); T1, T2 and T3: first (n = 69), second (n = 81) and third (n = 77) trimester of pregnancy, respectively.

The mean intake of energy changed from PC to late pregnancy (p < 0.0001; Table 2). Similar variations in the estimated energy requirements were observed during PC and throughout pregnancy. When the energy intakes of each participant were compared with their respective estimated energy requirement, the mean energy intakes were within one standard error of the predicted value of the estimated energy requirement for 33.3% of the participants in PC, 42.0% in T1, 53.1% in T2 and 39.0% in T3 (Figure 2). Excluding individuals who were breastfeeding during the PC period did not significantly change the findings, except for energy intake. The latter still increased from PC to T3 (p < 0.0001), but it became stable during pregnancy; the increase from T1 to T3 changed from +150 kcal/day (p = 0.02) to +144 kcal/day (p = 0.06).

**Table 2.** Energy and macronutrient intakes in preconception and over the course of pregnancy.

Variables	Mean ± SD or n (%)				Overall p-value
	PC (n = 90)	T1 (n = 71)	T2 (n = 83)	T3 (n = 78)	
Energy intake (kcal/day)	2172.3 ± 456.8^b^	2283.9 ± 556.8^b,c^	2381.6 ± 500.5^c,d^	2434.0 ± 548.9^d^	**<0.0001**
Estimated energy requirements^a^ (kcal/day)	2156.1 ± 228.5^b^	2158.1 ± 232.4^b^	2489.9 ± 207.0^c^	2560.1 ± 231.5^c^	**<0.0001**
Macronutrient intakes					
Carbohydrates (g/day)	247.7 ± 61.5^b^	279.4 ± 74.5^c^	291.3 ± 71.0^c^	299.9 ± 73.3^c^	**<0.0001**
%EI	44.0 ± 6.2^b^	47.2 ± 5.6^c^	47.0 ± 5.5^c^	47.5 ± 6.1^c^	**<0.0001**
Dietary fibers (g/day)	23.3 ± 9.0^b^	24.8 ± 9.1^b,c^	26.4 ± 10.0^c^	27.2 ± 9.4^c^	**0.0001**
Dietary fibers (g/1000 kcal/day)	10.8 ± 3.4	11.0 ± 3.2	11.1 ± 3.3	11.2 ± 3.2	0.35
<14	80 (88.9)	60 (84.5)	67 (80.7)	67 (85.9)	0.06
Total fats (g/day)	88.9 ± 23.0^b^	92.1 ± 27.0^b,c^	97.3 ± 25.9^c,d^	99.1 ± 28.9^d^	**0.001**
%EI	36.8 ± 5.0	36.3 ± 4.6	36.6 ± 4.6	36.5 ± 5.1	0.61
Proteins (g/day)	86.9 ± 22.8^b^	93.1 ± 24.4^b,c^	95.9 ± 22.9^c^	96.5 ± 26.0^c^	**0.0009**
Estimated protein requirements (g/day)	40.9 ± 4.7^b^	41.2 ± 5.0^b^	54.3 ± 6.4^c^	54.3 ± 6.5^c^	**<0.0001**
< Estimated protein requirements	2 (2.2)	0 (0.0)	1 (1.2)	3 (3.8)	0.05
%EI	16.0 ± 2.8	16.4 ± 2.7	16.2 ± 2.8	15.9 ± 2.3	0.56
Alcohol (g/day)	10.6 ± 16.2^b^	0.1 ± 0.6^c^	0.3 ± 2.0^c^	0.3 ± 1.1^c^	**<0.0001**
%EI	3.2 ± 4.6^b^	0.1 ± 0.2^c^	0.1 ± 0.6^c^	0.1 ± 0.3^c^	**<0.0001**

Values with different superscript letters (b, c, d) are different at p < 0.05 (after Bonferroni correction when applicable). Values in bold represent statistically significant differences (p < 0.05).

%EI: percentage of contribution to daily energy intake; IU: international unit; PC: preconception; T1, T2 and T3: first, second and third trimester of pregnancy, respectively.

a T1: n = 69, T2: n = 81, T3: n = 77.

#### Macronutrients intakes

The daily intakes of macronutrients are presented in Table 2. The mean intakes of carbohydrates, total fat proteins and dietary fibers increased from PC to late pregnancy (p < 0.01). The proportion of individuals below 14 g/1000 kcal for dietary fiber was stable over time (PC: 88.9%; T1: 84.5%; T2: 80.7%; T3: 85.9%, p = 0.06). Most participants had carbohydrate intakes below the acceptable macronutrient distribution range at PC (56.7%) and within the acceptable macronutrient distribution range in each trimester of pregnancy (T1: 63.3%; T2: 65.1%; T3: 64.1%; data not shown). In addition, the majority of participants had mean protein intakes within the acceptable macronutrient distribution range, whereas over 60.0% exceeded the acceptable macronutrient distribution range for total fat at each assessment (data not shown). The mean intakes of alcohol significantly changed over time (p < 0.0001); the intakes at PC were greater than the intakes at T1, T2 and T3 (p < 0.0001).

#### Micronutrient intakes

Data on micronutrient intakes from food sources and supplements as well as dietary supplement use are detailed in Table 3. Recommendations on the use of a multivitamin containing folic acid were met by a minority of participants before pregnancy, while most participants met the recommendations of consuming a multivitamin containing folic acid and iron during pregnancy (PC: 43.3%; T1: 67.1%; T2: 74.7%; T3: 69.5%, p < 0.0001; data not shown).

**Table 3.** Micronutrient intakes in preconception and over the course of pregnancy.

Variables	Mean ± SD or n (%)				Overall p-value
	PC (n = 90)	T1 (n = 71)	T2 (n = 83)	T3 (n = 78)	
Micronutrient intakes (from foods)					
Folic acid (µg/day)	113.7 ± 64.9^c^	143.6 ± 85.2^d^	143.4 ± 53.0^d^	140.1 ± 73.6^d^	**0.0001**
Folate (DFE/day)	488.8 ± 146.2^c^	515.2 ± 186.3^c,d^	540.6 ± 143.5^d^	551.0 ± 183.3^d^	**0.004**
EAR	9 (10.0)^c^	41 (57.8)^d^	41 (49.4)^d^	43 (55.1)^d^	**<0.0001**
Iron (mg/day)	14.0 ± 4.2^c^	14.9 ± 4.6^c,d^	16.0 ± 5.1^d,e^	16.5 ± 5.1^e^	**<0.0001**
EAR	3 (3.3)^c^	67 (94.4)^d^	76 (91.6)^d^	68 (87.2)^d^	**<0.0001**
>UL	0 (0.0)	0 (0.0)	0 (0.0)	0 (0.0)	
Vitamin D (IU/day)	213.6 ± 135.7	218.9 ± 135.2	240.4 ± 138.8	247.5 ± 137.5	0.08
EAR	83 (92.2)	61 (85.9)	73 (88.0)	67 (85.9)	0.20
>UL	0 (0.0)	0 (0.0)	0 (0.0)	0 (0.0)	
Vitamin B12 (µg/day)	5.1 ± 3.5	4.7 ± 2.7	4.8 ± 2.1	5.0 ± 2.3	0.47
EAR	7 (7.8)^c^	14 (19.7)^d^	3 (3.6)^c^	7 (9.0)^c,d^	**<0.0001**
Total micronutrient intakes (from foods and supplements)					
Total folic acid (µg/day)	846.5 ± 807.1^c^	1162.1 ± 821.7^d^	999.4 ± 598.3^d^	926.6 ± 650.6^d^	**<0.0001**
>UL	49 (54.4)^c^	57 (80.3)^d^	61 (73.5)^d^	49 (62.8)^c,d^	**<0.0001**
Total folate (DFE/day)	1710.2 ± 1359.9^c^	2212.8 ± 1388.6^d^	1967.2 ± 993.3^d^	1861.9 ± 1082.3^c,d^	**0.0002**
EAR	3 (3.3)^c^	5 (7.0)^c,d^	7 (8.4)^c,d^	11 (14.1)^d^	**0.002**
Total iron (mg/day)	23.4 ± 13.6^c^	33.6 ± 14.6^d^	40.6 ± 28.4^d^	44.3 ± 33.0^d^	**<0.0001**
EAR	2 (2.2)^c^	21 (29.6)^d^	18 (21.7)^d^	18 (23.1)^d^	**<0.0001**
>UL	7 (7.8)^c^	14 (19.7)^c,d^	22 (26.5)^d^	29 (37.2)^d^	**<0.0001**
Total vitamin D (IU/day)	517.9 ± 391.3^c^	656.9 ± 389.6^d^	659.3 ± 336.5^d^	674.7 ± 354.0^d^	**<0.0001**
EAR	42 (46.7)^c^	14 (19.7)^d^	15 (18.1)^d^	17 (21.8)^d^	**<0.0001**
>UL	11 (12.2)	9 (12.7)	8 (9.6)	13 (16.7)	0.15
Total vitamin B12 (µg/day)	24.9 ± 109.5	27.9 ± 100.2	28.6 ± 100.6	33.4 ± 110.4	0.54
EAR	1 (1.1)	4 (5.6)	1 (1.2)	1 (1.3)	0.05
Supplement use^a^					
None^b^	25 (27.8)^c^	7 (9.6)^d^	11 (12.6)^d^	17 (20.7)^c,d^	**0.0001**
Multivitamin alone	35 (38.9)^c^	49 (67.1)^d^	68 (78.2)^d^	62 (75.6)^d^	**<0.0001**
Folic acid alone	26 (28.9)^c^	12 (16.4)^c^	8 (9.2)^d^	3 (3.7)^d^	**<0.0001**
Multivitamin and folic acid	4 (4.4)	5 (6.9)	0 (0.0)	0 (0.0)	**0.0003**

Values with different superscript letters (c, d, e) are different at p < 0.05 (after Bonferroni correction when applicable). Values in bold represent statistically significant differences (p < 0.05).

DFE: dietary folate equivalents; EAR: estimated average requirement; IU: international unit; PC: preconception; T1, T2 and T3: first, second and third trimester of pregnancy, respectively; UL: tolerable upper intake level.

a T1: n = 73, T2: n = 87, T3: n = 82.

b No folic acid vitamin nor multivitamin.

With respect to micronutrient intakes from food sources and supplements, the mean total intakes of folate changed over time (p = 0.0002), especially from PC to early pregnancy (+502.6 DFE/day, p = 0.0004). The proportion of participants with mean total intakes of folate below the EAR was greater at T3 than at PC (+10.8%, p = 0.02). The proportion of participants with a mean total intake of folic acid above the UL increased from PC to T1 (+25.9%, p = 0.006) and remained statistically stable throughout pregnancy. The mean total folic acid intake exceeded that of the UL by a mean of 308 µg/day in PC, 364 µg/day in T1, 222 µg/day in T2 and 259 µg/day in T3 (data not shown). Folic acid intake from supplements represented 63%–78% of the folic acid mean total intake in both periods (data not shown). The mean total intake of iron increased over time (p < 0.0001), notably from PC to T1 (+10.2 mg/day, p < 0.0001). Nevertheless, more than 20% of the participants still had iron mean total intakes below the EAR in each trimester. The mean total intake of vitamin D also increased over time (p < 0.0001), especially from PC to T3 (+156.8 IU/day, p = 0.009). Nearly half of the participants had vitamin D mean total intakes below the EAR at PC, whereas fewer than a quarter had vitamin D mean total intakes below the EAR during each trimester. The mean total intake of vitamin B12 was similar before and during pregnancy and above the EAR for each measurement period for almost all participants (PC: 98.9%, T1: 94.4%, T2: 98.8%, T3: 98.7%).

### Diet quality

**Figure 3.**
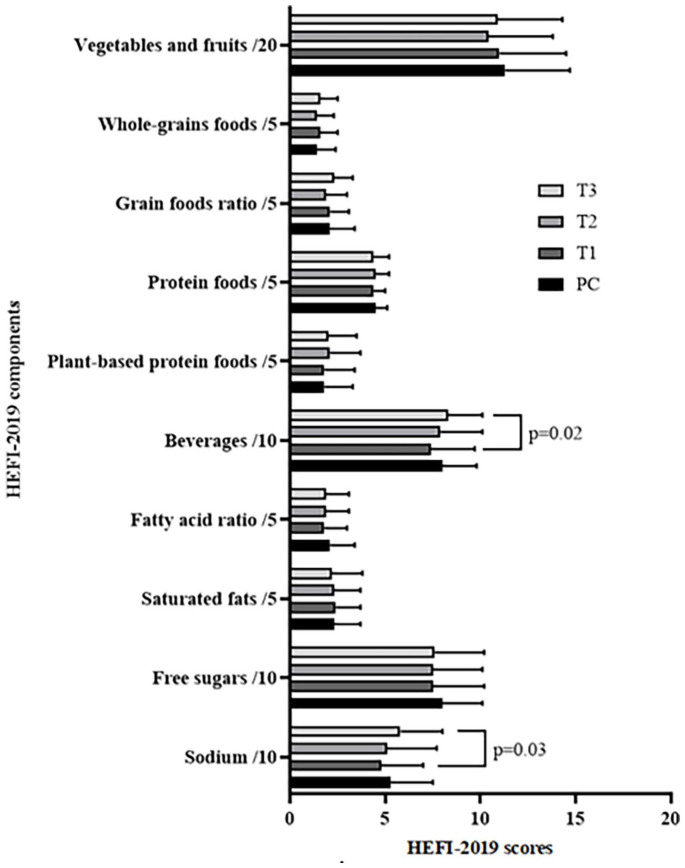
Scores of each HEFI-2019 component in PC and in each trimester of pregnancy. HEFI-2019: Healthy Eating Food Index 2019; PC: preconception (n = 90); T1, T2 and T3: first (n = 72), second (n = 83) and third (n = 78) trimester of pregnancy, respectively.

No change in the HEFI-2019 total score was observed from PC to T3 (PC: 46.9 ± 8.5; T1: 44.9 ± 8.7; T2: 45.0 ± 8.9; T3: 47.0 ± 8.4, p = 0.10). In terms of specific HEFI-2019 components (Figure 3), there was an increase in the beverage component score from T1 to T3 (+0.85/10, p = 0.02), indicating that there were proportionally more water and unsweetened beverages consumed than all beverages consumed in T3 than in T1. An increase in the sodium component score was observed from T1 to T3 (+0.99/10, p = 0.03), indicating that less sodium was consumed in proportion to total energy intake in T3 than in T1.

### Physical activity levels

**Figure 4.**
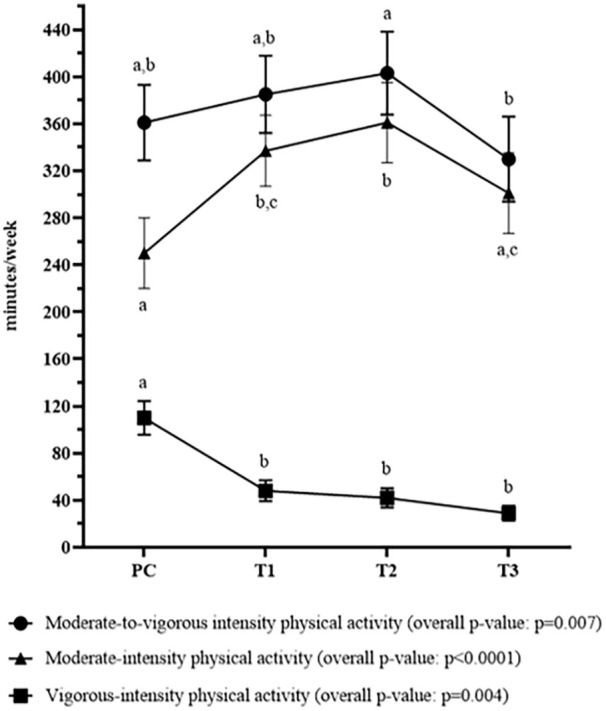
Physical activity levels in preconception and in each trimester of pregnancy. Values or cells with different letters indicate that the time spent in moderate and/or vigorous intensity physical activity for a time point is different than another time point at p < 0.05 (after Bonferroni correction when applicable). Error bars represent the standard error of the mean. PC: preconception (n = 90); T1, T2 and T3: first (n = 72), second (n = 83) and third (n = 78) trimester of pregnancy, respectively.

Significant changes in physical activity levels were observed across the PC and pregnancy periods. More specifically, the time spent per week in moderate-intensity physical activity increased from PC to T1 (p = 0.002), remained similar from T1 to T2 (p = 0.85) and decreased from T2 to T3 (p = 0.02; Figure 4). The time spent in vigorous-intensity decreased from PC to T1 (p = 0.005) and then remained stable from T1 to T3 (p > 0.05). When the time spent at moderate and vigorous intensities was combined, physical activity levels significantly changed over time (p = 0.007); they were stable from PC to T1 (p > 0.05), remained statistically similar from T1 to T2 (p = 0.79) and then decreased from T2 to T3 to lower levels (p = 0.004). The physical activity volume (expressed in METs – min/week) and the proportion of participants reaching physical activity levels meeting national guidelines^37,38^ followed a similar pattern (Table 4). Both were lower at T3 than at all other time points (p < 0.05), with the most significant decrease observed between PC and T3 (−496 METs – min/week, p ⩽ 0.0001 and −18% of people meeting guidelines, p = 0.01).

**Table 4.** Physical activity levels in preconception and over the course of pregnancy.

Physical activity levels	Mean ± SD or n (%)				
	PC (n = 90)	T1 (n = 71)	T2 (n = 85)	T3 (n = 81)	Overall p-value
Total METs – min/week	1754 ± 1431^a^	1518 ± 1124^a^	1562 ± 1214^a^	1258 ± 1218^b^	**0.0001**
Meeting physical activity guidelines	74 (82.2)^a^	57 (80.3)^a^	66 (77.6)^a^	52 (64.2)^b^	<**0.0001**

Total METs – min/week was obtained from the sum of METs – min/week devoted to all physical activities of moderate or vigorous intensity (walking, sports and leisure) across all physical activity categories. Physical activity guidelines refer to the achievement of at least 150 min/week of moderate-to-vigorous intensity physical activity in preconception and at least 150 min/week of moderate intensity physical activity during pregnancy. Values or cells with different letters (a,b) indicate that the total METs – min/week or the proportions of participants in each physical activity categories for a time point are different than another time point at p < 0.05 (after Bonferroni correction when applicable). Values in bold represent statistically significant differences (p < 0.05).

PC: preconception; T1, T2 and T3: first, second and third trimester of pregnancy, respectively.

## Discussion

To our knowledge, this is the first study to prospectively evaluate the intakes of energy, macronutrients and specific micronutrients; diet quality; and physical activity levels in PC and throughout pregnancy. Energy intakes increased while diet quality remained stable across those time points. Although intakes of dietary fibers increased from PC to late pregnancy, more than 80% of individuals were below adequate dietary fiber intake in both periods. Total intakes of micronutrients (from food sources and supplements), including folate, folic acid, iron and vitamin D, increased from PC to early pregnancy and remained stable during pregnancy. Micronutrient total intakes primarily came from supplements and met the needs for folate, iron and vitamins D and B12 in PC and throughout pregnancy for at least half of participants. The volume of leisure-time and sports activities was relatively stable from PC to mid-pregnancy and then markedly decreased from mid- to late pregnancy. Overall, more than 60% of the individuals met the Canadian physical activity guidelines.

### Nutrition and physical activity from PC to early pregnancy

Contrary to our hypotheses, no change was observed from PC to the first trimester in some studied components, such as energy intake, diet quality, physical activity volume (i.e. METs – min/week) and the proportion of individuals meeting the Canadian Guidelines for physical activity. This stability can be partly explained by the fact that changing a behavior is difficult and can last long without adequate social support and sufficient knowledge.^39^ Moreover, there is no systematic intervention targeting eating and physical activity behaviors during the PC period or in early pregnancy. In our study, a high proportion of individuals planning a pregnancy (89%) had intakes of dietary fibers below their estimated needs, which is consistent with few other studies conducted among women of childbearing age.^40–42^ This high proportion remained similar throughout pregnancy. The difference between the intake consumed and the recommended intake varied from 2.8 to 3.2 g/1000 kcal which represents a considerable gap. Adequate intakes of dietary fibers in PC can have protective effects on the risk of developing chronic diseases (i.e. cardiovascular disease, type 2 diabetes and some cancers)^43^ and has potential implications in the prevention of hypertensive disorders of pregnancy and preterm birth.^44,45^ Future interventions could consider including a specific component of dietary fibers to detect insufficiency before the onset of pregnancy or as early as possible during pregnancy.

In our study, physical activity volume may have not changed from PC to early pregnancy, but we found a significant increase in moderate-intensity activities and a marked decrease in vigorous-intensity activities, suggesting that individuals continued their physical activity once pregnant but replaced some of their vigorous-intensity activities with moderate-intensity activities. Importantly, individuals were already quite active before pregnancy and in the first trimester, with 82% and 80%, respectively, meeting the guidelines. This result suggests a certain motivation to maintain physical activity levels even during pregnancy and aligns with the fact that prepregnancy physical activity is the strongest predictor of physical activity levels during pregnancy.^20,46^

In line with our assumption, the mean total intakes (from foods and supplements) of folate, folic acid, iron and vitamin D in PC were lower than in pregnancy, which can be attributed to a greater proportion of participants who met supplement use recommendations during pregnancy than in PC (67%–75% versus 43%). Studies reporting the proportion of multivitamin users before and/or during pregnancy have shown similar^47–49^ or higher (>80% in PC and >90% in pregnancy)^50,51^ proportions. When comparing the intakes of micronutrients from food sources to the dietary reference intakes, most individuals had intakes below the EAR for vitamin D in PC. These results are supported by cross-sectional PC cohort studies.^42,52^ When food sources and dietary supplements are combined, 47% of individuals planning a pregnancy still have insufficient vitamin D intakes. This result is in accordance with those of a few other studies.^10,48^ The proportion of individuals planning a pregnancy with total vitamin D intakes below the requirements fell by half once pregnant, from 47% in PC to 20% in early pregnancy, mainly due to a significant increase in vitamin D intake from supplements, which is an important determinant of vitamin D status.^53^ With respect to folic acid, the results revealed that 54% of the participants had mean total intakes above the UL before conception and that once pregnant, the percentage above the UL increased considerably to 80%. Overall, the results support the importance of supplementation in Canada during PC, especially for vitamin D. Our findings also emphasize the importance of consuming 400 µg of folic acid in both PC and pregnancy, not more, unless a specific condition is denoted, such as having had a previous pregnancy with neural tube defects, per example.^54^

### Nutrition and physical activity from early to late pregnancy

We expected stable energy intake during pregnancy, as reported in a systematic review and meta-analysis^13^ as well as in other studies conducted subsequently^12,14^; however, we found an increase from early to late pregnancy. This energy intake pattern observed in our study is aligned with the increased needs during pregnancy, as stated in dietary recommendations.^15^ A recent systematic review and meta-analysis revealed greater energy intakes at T3 than at T1 and T2.^55^ These data should be interpreted carefully since the overall heterogeneity of the studies included in the meta-analysis was high (heterogeneity coefficient (*I*^2^) = 99.88%). Therefore, no conclusion can be drawn about the trajectory of energy intake during pregnancy. The lack of consensus in energy intake patterns during pregnancy (i.e. stability versus increase) reported in the literature may be explained by multiple factors, such as the method use to assess energy intakes and interindividual variabilities, including body weight, dieting behaviors, energy expenditure related to basal metabolism and physical activity and hormonal status. These factors can influence energy intakes^56^ and have the potential to evolve differently over time from one individual to another.

We also observed a stable HEFI-2019 total score throughout pregnancy. Individuals in our study had HEFI-2019 total scores before conception and throughout pregnancy that were similar to those of Canadian individuals in the general population (43.1/80), reflecting a relatively low degree of adherence to the 2019 Canada’s Food Guide.^30^ Mixed findings have been reported in the literature regarding prenatal diet quality patterns. Few studies have shown stability from early to late pregnancy,^57,58^ whereas few others have shown a decrease in diet quality from early to mid-pregnancy or an inverted U-shaped pattern from early to late pregnancy.^59,60^ Discrepancies in results may be partly due to the tools used to assess dietary intakes and derive diet quality scores. For example, different food components are included in the wide variety of diet quality scores available in the literature.^57–59^ Inter- and intraindividual variabilities may also have contributed to the mixed findings.^61^ Kant and Graubard reported that some sociodemographic characteristics likely contributed to the variations found in the quality of diets.^61^ Although micronutrients are not included in diet quality scores, they are important nutrients required for a healthy diet, especially during PC and pregnancy.^28^

When micronutrients from food sources were compared with dietary reference intakes, most pregnant individuals had intakes below the EAR for vitamin D, folate and iron intakes in each trimester. These results are corroborated by those of cross-sectional^62,63^ and prospective^14,64^ pregnancy cohort studies. When combining intakes from food sources and dietary supplements, we found a considerable decrease in the proportion of pregnant individuals with vitamin D and iron intakes below the EAR; approximately three pregnant individuals out of four had vitamin D and total iron intakes above the EAR, whereas the remaining 25% were not far from reaching the recommendations. With respect to vitamin B9, the results showed that most participants had total intakes of folate above the EAR and total intakes of folic acid above the UL throughout pregnancy, which is consistent with other studies conducted among pregnant individuals.^14,50,62,65,66^ These findings support the importance of dietary supplementation during pregnancy and highlight the importance of a systematic follow-up of the doses taken in dietary supplements during prenatal visits. These results also underscore the importance of validating micronutrient status via biochemical measurements in individuals with low intakes and thus at high risk of deficiency.

Finally, the physical activity volume (i.e. METs – min/week) and the proportion of individuals meeting the Canadian Guidelines for Physical Activity were statistically stable from early to mid-pregnancy and then markedly decreased at T3. This decrease in physical activity levels at T3 could be explained by increased shortness of breath during the last months of pregnancy or by lumbopelvic pain, which generally increases in prevalence and severity as pregnancy progresses.^67,68^

### Strengths and limitations

This study has several strengths. First, the prospective evaluation of dietary intakes, diet quality and physical activity levels in the same individuals throughout two important and challenging periods allowed the collection of extensive and robust data. The fact that nutrition and physical activity data were collected during each trimester in the same individual also allowed a better understanding of how these prenatal lifestyle habits evolve across two important periods of life. Second, dietary intakes were evaluated in PC and during each trimester using at least two R24W per assessment. Although this assessment remains imperfect, it provided a broad picture of nutrient consumption, attenuated random errors such as intraindividual variations and increased the precision of estimates compared to the use of a single R24W.^69^ Third, micronutrient intakes from foods and supplements were thoroughly assessed to identify the proportion of individuals with adequate or inadequate intakes before and during pregnancy.

Some limitations should be acknowledged. Our population was homogenous in terms of socioeconomic and demographic characteristics (e.g. Caucasian, well-educated, high income, etc.), limiting the generalizability of our findings. The recruitment strategy included social media and university outreach which may have led to selection bias favoring more health-conscious individuals. The sample size was relatively small and no power analysis for sample size calculation was done. This may have affected the detection of statistically significant changes. We also used self-administered questionnaires, which may have led to under- or overestimation of nutritional and physical activity data. The dietary intakes reported in the present study are derived from multiple R24W at each time point, leading to mean intakes rather than participants’ usual intakes. Nutritional data may have been biased by the fact that 12.9% of our sample were breastfeeding at the time of their PC assessment. We documented the presence or absence of breastfeeding during PC, but we do not know how long these people had been breastfeeding or the frequency and duration of feeding. These factors can influence individuals’ nutritional needs, dietary intakes, diet quality and even physical activity levels.^70^ However, when removing individuals who breastfed at PC, changes in results were minor. Another limitation is that the HEFI-2019 has never been validated in a pregnant population. This index still accurately reflects adherence to the most recent healthy eating recommendations of Canada’s food guide, which is also recommended during pregnancy. Finally, the use of two different physical activity questionnaires may have limited the comparability of physical activity data collected before and during pregnancy. Even so, both questionnaires have been correlated,^71,72^ harmonized in terms of METs attributed to moderate- and vigorous-intensity physical activity and validated^32,34,35^ in the population for which they were used in this study.

## Conclusion

In conclusion, individuals planning a pregnancy in the province of Quebec (Canada) who became pregnant within the next year presented increases in energy, macronutrients and specific micronutrients intakes from PC to pregnancy. However, no change was found in their diet quality. While a majority of individuals reached the recommendations for the micronutrients studied, largely due to supplementation during pregnancy, a high proportion of individuals had dietary fiber intakes below the recommendations in PC and throughout pregnancy. Physical activity levels remained relatively stable from PC to mid-pregnancy and then decreased in late pregnancy. Although these results need to be confirmed in a larger, more heterogeneous sample, and that most recommendations are met for the majority of participants, we can nevertheless state that individuals of reproductive age could benefit from improving their diet and maintaining adequate physical activity levels. Future studies are needed to determine the best course of action to support individuals aiming to improve and maintain their health behaviors on a continuum from PC to the end of pregnancy.

## Supplemental Material

sj-docx-1-whe-10.1177_17455057251341999 – Supplemental material for Dietary intakes, diet quality and physical activity levels from preconception to late pregnancy: Prospective assessment of changes and adherence to recommendations Supplemental material, sj-docx-1-whe-10.1177_17455057251341999 for Dietary intakes, diet quality and physical activity levels from preconception to late pregnancy: Prospective assessment of changes and adherence to recommendations by Audrey St-Laurent, Anne-Sophie Plante, Stéphanie Harrison, Simone Lemieux, Julie Robitaille, Stephanie-May Ruchat and Anne-Sophie Morisset in Women’s Health

sj-pdf-2-whe-10.1177_17455057251341999 – Supplemental material for Dietary intakes, diet quality and physical activity levels from preconception to late pregnancy: Prospective assessment of changes and adherence to recommendations Supplemental material, sj-pdf-2-whe-10.1177_17455057251341999 for Dietary intakes, diet quality and physical activity levels from preconception to late pregnancy: Prospective assessment of changes and adherence to recommendations by Audrey St-Laurent, Anne-Sophie Plante, Stéphanie Harrison, Simone Lemieux, Julie Robitaille, Stephanie-May Ruchat and Anne-Sophie Morisset in Women’s Health
