# Supplementary material for: Dietary intakes, diet quality and physical activity levels from preconception to late pregnancy: Prospective assessment of changes and adherence to recommendations
Source: Womens Health (Lond). 2025 Jun 24;21:17455057251341999. doi: 10.1177/17455057251341999 (PMC12188061; doi:10.1177/17455057251341999)
Supplement: sj-pdf-2-whe-10.1177_17455057251341999 – Supplemental material for Dietary intakes, diet quality and physical activity levels from preconception to late pregnancy: Prospective assessment of changes and adherence to recommendations [file sj-pdf-2-whe-10.1177_17455057251341999.pdf]

Supplementary materials 2-A. General health questionnaire

ÉTAT DE SANTÉ AVANT LA GROSSESSE

1. Date de naissance?

(aaaa-mm-jj)

2. Combien pesez-vous actuellement?

livres **OU**

kilogrammes

3. Combien mesurez-vous?

pieds **ET** pouces **OU**

mètres

4. Consommez-vous du tabac actuellement?

○ Oui ○ Non

Si oui, combien par jour? cigarettes/jour

Date d'arrêt, s'il y a lieu

Histoire de poids

5. Quel est votre poids habituel (c'est-à-dire le poids ayant été maintenu le plus longtemps à l'âge adulte, en excluant la grossesse ou les grossesses précédentes)?

Kg ou lbs ○ Ne sais pas

6. Quel était votre poids à l'âge de 25 ans?

kg ou lbs ○ ne sais pas ○ ne s'applique pas

7. Avant votre grossesse, vous considériez-vous comme étant préoccupée par votre apparence et/ou par votre poids?

○ Oui

○ Non

○ Ne sais pas

Grossesse, allaitement, menstruations

8. Nombre d'enfant(s):

[ ] enfant(s)

9. Quel était votre âge lors de la grossesse de votre premier enfant:

[ ] ans

10. Nombre de grossesse(s) - incluant votre grossesse actuelle:

[ ] grossesse(s)

		Oui	Nombre	Non
**11.**	Fausse couche:	○	[ ]	○
**12.**	Avortement:	○	[ ]	○

13. Avez-vous déjà souffert de diabète de grossesse lors d'une ou plusieurs de vos grossesses ?

○ Oui

○ Non

					Si oui, précisez à quelle(s) grossesse(s)
**14.**	Avez-vous reçu un suivi médical lors de votre diabète de grossesse?	○ Oui	○ Non	○ Ne s'applique pas	[ ]
**15.**	Avez-vous reçu un suivi nutritionnel (avec nutritionniste) lors de votre diabète de grossesse?	○ Oui	○ Non	○ Ne s'applique pas	[ ]

16. Avez-vous pris des médicaments pour traiter votre diabète de grossesse?

○ Oui ○ Non ○ Ne s'applique pas

Si oui, précisez à quelle(s) grossesse(s) ________

Si oui, le type de médicaments (hypoglycémiants, insuline ou autres) ________

17. Allaitez-vous présentement ?

○ Oui ○ Non

18. Avez-vous déjà allaité?

○ Oui ○ Non

19. Nombre de bébé(s) allaité(s)?

________ bébé(s)

20. Durée moyenne de l'allaitement pour chaque enfant:

1er enfant ________ mois
2e enfant ________ mois
3e enfant ________ mois
4e enfant ________ mois
Autre(s) enfant(s) ________ mois

21. Avez-vous déjà pris des contraceptifs oraux par le passé?

○ Oui ○ Non

Si oui, date d'arrêt: ________

Si oui, type de contraceptif: ________

22. À quel âge avez-vous eu vos premières menstruations?

________ ans ○ Je ne sais pas

23. Aviez-vous un cycle menstruel régulier avant votre grossesse?

○ Oui

○ Non

○ Je ne sais pas

24. Durée du cycle menstruel:

[ ] jours ○ Ne sait pas

25. Date des dernières menstruations (1re journée):

[ ] ○ Ne sait pas

Histoire familiale et histoire personnelle

26. Histoire familiale ⓘ

	Oui	Non	Ne sais pas	Si oui, préciser
Diabète de type 1	○	○	○	
Diabète de type 2	○	○	○	
Maladies cardiovasculaires (classe 1) ⓘ	○	○	○	
Maladies cardiovasculaires (classe 2) ⓘ	○	○	○	
Hypertension	○	○	○	
Dyslipidémie	○	○	○	
Désordres endocriniens	○	○	○	
Cancers	○	○	○	
Autres	○	○	○	

27. Histoire personnelle

	Oui	Non	Ne sait pas	Si oui, préciser:	Âge au diagnostic
Diabète de type 1	○	○	○		ans
Diabète de type 2	○	○	○		ans
Maladies cardiovasculaires (classe 1) ⓘ	○	○	○		ans
Maladies cardiovasculaires (classe 2) ⓘ	○	○	○		ans
Hypertension	○	○	○		ans
Dyslipidémies	○	○	○		ans
Troubles endocriniens ⓘ	○	○	○		ans
Troubles gastrointestinaux ⓘ	○	○	○		ans
Maladies hépatiques (foie) ⓘ	○	○	○		ans
Cancers ⓘ	○	○	○		ans
Chirurgie(s)	○	○	○		ans
Autre(s)	○	○	○		ans
Maladies rénales ⓘ	○	○	○		ans

28.

La médication durant la grossesse

Prenez-vous de la médication présentement?

○ Oui ○ Non

Nom du médicament	Dose	Fréquence	Indication	Début	Arrêt	*
						☐
						☐
						☐
						☐
						☐
						☐
						☐

Avez-vous des allergies à un/des médicament(s)? Oui ○ Non ○ Si oui, spécifier:

Allergies, habitudes alimentaires, consommation d'aliments spécifiques

	Oui	Non	Ne sait pas	Si oui, spécifier:
**29.** Avez-vous des allergies alimentaires?	○	○	○	
**30.** Avez-vous d'autres types d'allergies ?	○	○	○	
**31.** Avez-vous des intolérances alimentaires?	○	○	○	
**32.** Avez-vous des habitudes alimentaires particulières (ex. végétarisme) ?	○	○	○	

33. Combien de repas par jour consommez-vous?

_____ repas

34. Combien de collation(s) par jour consommez-vous?

[ ] collation(s)

35. En général, qui prépare(nt) les repas à la maison?

- ○ Moi
- ○ Mon ou ma conjoint(e)
- ○ Les deux
- ○ Autre membre de la famille
- ○ Autre personne

36. Partagez-vous habituellement vos repas avec les autres membres de la famille?

Oui	Non
○	○

37. Vous arrive-t-il de fréquenter les restaurants?

- ○ Jamais
- ○ 1 à 3 fois par mois
- ○ 1 fois/semaine
- ○ 2 à 4 fois par semaine
- ○ 5 à 6 fois par semaine
- ○ 1 fois et plus par jour

38. Quel type de restaurants choisissez-vous le plus souvent?

- ○ Familial (par exemple: Normandin, St-Hubert, etc.)
- ○ Service rapide (par exemple: McDonald's, Burger King, etc.)
- ○ Exotique (par exemple: sushi bar, asiatique, etc.)
- ○ Spécialisé (par exemple: steak-house, poissons et fruits de mer, etc.)
- ○ Café-restaurant (par exemple: sandwicherie, soupes et salades, etc.)
- ○ Cafétéria ou libre-service (par exemple: bar à salade, mets à emporter, etc.)

Supplementary materials 2-B. Sociodemographic questionnaire

QUESTIONNAIRE SOCIO-ÉCONOMIQUE ET DÉMOGRAPHIQUE

DIRECTIVES: Lire attentivement les questions. Les réponses à ce questionnaire vont demeurer confidentielles.

Toutes les réponses sont importantes pour les chercheurs, toutetefois, les participants peuvent choisir d'y répondre complètement ou partiellement.

1. Quelle est votre langue maternelle?

- ○ Français
- ○ Anglais
- ○ Autre

SVP précisez: ______________________

2. De quelle origine ethnique/culturelle êtes-vous? (le masculin a été utilisé pour ne pas alourdir le texte.)

- ○ Blanc (Ex. Canada, États-Unis, Europe, Moyen-Orient, Afrique du Nord, etc.)
- ○ Noir (Ex. Jamaïque, Caraïbes, Niger, Haïti, etc.)
- ○ Autochtone
- ○ Inuit
- ○ Asiatique (Ex. Extrême-Orient, Asie du Sud-Est, Cambodge, Chine, etc.)
- ○ Hispanique (Ex. Mexique, Porto Rico, Cuba, République Dominicaine, etc.)
- ○ Je préfère ne pas répondre
- ○ Autre

Préciser: ______________________

3. Quel est votre état matrimonial actuel?

- ○ Marié(e)
- ○ Divorcé(e)
- ○ Union libre
- ○ Veuf(ve)
- ○ Séparé(e)
- ○ Célibataire
- ○ Je préfère ne pas répondre

4. Avez-vous des enfants?

○ Oui ○ Non ○ Je préfère ne pas répondre

5. Combien avez-vous d'enfants?

1	2	3	4 et plus	Je préfère ne pas répondre
○	○	○	○	○

6. Quel âge ont-ils?

[ ] ⓘ

7. Avec qui habitez-vous?

- ☐ Conjoint(e)
- ☐ Conjoint(e) et enfant(s)
- ☐ Un ou des enfant(s)
- ☐ D'autres personnes (Ex. parent, colocataire, etc.)
- ☐ Seul(e)
- ☐ Je préfère ne pas répondre

8. Quel est le type de milieu où vous habitez?

- ○ Urbain (en ville)
- ○ Rural (en campagne)
- ○ Banlieue
- ○ Je préfère ne pas répondre

9. Cochez la ou les situation(s) qui s'applique(nt) à vous :

- ☐ Étudiant(e) à temps plein
- ☐ Étudiant (e) à temps partiel
- ☐ Travailleur (euse) à temps plein
- ☐ Travailleur (euse) à temps partiel
- ☐ Travailleur(euse) saisonnier
- ☐ Travailleur(euse) autonome
- ☐ Chômeur(euse)/sans emploi
- ☐ Parent au foyer
- ☐ Retraité(e)
- ☐ Inapte au travail
- ☐ Autre
- ☐ Je préfère ne pas répondre

10. Quel est votre emploi actuel (ou quel était votre dernier emploi si vous ne travaillez pas actuellement)? ⓘ

____

11. Quel est votre type d'horaire de travail (ou quel était-il)?

- ○ Heures régulières de jour
- ○ Heures régulières de soir
- ○ Heures régulières de nuit
- ○ Quarts de travail de jour et/ou de nuit
- ○ Heures irrégulières
- ○ Autre
- ○ Je préfère ne pas répondre

12. Quel était approximativement votre revenu familial total l'an dernier avant déduction d'impôts ?

- ○ 0$ - 19 999$
- ○ 20 000$ - 39 999$
- ○ 40 000$ - 59 999$
- ○ 60 000$ - 79 999$
- ○ 80 000$ - 99 999$
- ○ 100 000$ et plus
- ○ Je préfère ne pas répondre

13. Quel est le plus haut niveau de scolarité que vous avez complété?

- ○ Aucune scolarité (ou niveau primaire non complété)
- ○ Primaire
- ○ Secondaire (Ex. secondaire 5, DEP, etc.)
- ○ Collégial (Ex. DEC général ou technique, AEC, etc.)
- ○ Universitaire (Ex. certificat, baccalauréat, maîtrise, doctorat, etc.)
- ○ Je préfère ne pas répondre

Supplementary materials 2-C. Questionnaire on supplement use

QUESTIONNAIRE SUR LES SUPPLÉMENTS ALIMENTAIRES

Suppléments alimentaires

1. Dans le dernier mois, avez-vous pris un supplément alimentaire (vitamine/minéraux)?
Attention: Considérez ***aussi*** *les suppléments alimentaires vendus sous la forme d'huiles (ex. huile de foie de morue).*

- ○ Oui
- ○ Non

2. Inscrivez le nom et le numéro d'identification de ce supplément.

Nom du supplément __________

Numéro d'identification du supplément (DIN) ⓘ __________

3. Avec quelle **unité de mesure** souhaitez-vous rapporter la dose de ce supplément?

- ○ ml
- ○ Goutte
- ○ Comprimé
- ○ Capsule
- ○ Cuillère à soupe
- ○ Cuillère à thé
- ○ Autre

Si vous avez choisi "*Autre*", veuillez **préciser**: __________

4. Inscrivez **la dose** de votre supplément selon l'unité de mesure choisie à la question précédente.
S'il s'agit d'une multivitamine, inscrivez le nombre de <u>*comprimés/capsules*</u>.

5. À quelle **fréquence** prenez-vous cette dose?

- ○ 1 fois par jour
- ○ 2 fois par jour
- ○ 3 fois par jour
- ○ Au besoin
- ○ Autre

Précisez :

6. Dans le dernier mois, avez-vous pris un **autre** supplément alimentaire (vitamine/minéraux)?
*Attention: Considérez **aussi** les suppléments alimentaires vendus sous la forme d'huiles (ex. huile de foie de morue).*

- ○ Oui
- ○ Non

7. Inscrivez le nom et le numéro d'identification de ce deuxième supplément.

Nom du supplément

Numéro d'identification du supplément (DIN)

8. Avec quelle **unité de mesure** souhaitez-vous rapporter la dose de ce deuxième supplément?

- ○ ml
- ○ Goutte
- ○ Comprimé
- ○ Capsule
- ○ Cuillère à soupe
- ○ Cuillère à thé
- ○ Autre

Si vous avez choisi "*Autre*", veuillez **préciser**: ______________________

9. Inscrivez **la dose** de ce deuxième supplément selon l'unité de mesure choisie à la question précédente.
S'il s'agit d'une multivitamine, inscrivez le nombre de <u>comprimés/capsules</u>.

__________ ⓘ

10. À quelle **fréquence** prenez-vous cette dose?

- ○ 1 fois par jour
- ○ 2 fois par jour
- ○ 3 fois par jour
- ○ Au besoin
- ○ Autre

Précisez : ______________________

11. Dans le dernier mois, avez-vous pris un **autre** supplément alimentaire (vitamine/minéraux)?
*Attention: Considérez **aussi** les suppléments alimentaires vendus sous la forme d'huiles (ex. huile de foie de morue).*

- ○ Oui
- ○ Non

12. Inscrivez le nom et le numéro d’identification de ce troisième supplément.

Nom du supplément

Numéro d'identification du supplément (DIN)

13. Avec quelle **unité de mesure** souhaitez-vous rapporter la dose de ce troisième supplément?

- ○ ml
- ○ Goutte
- ○ Comprimé
- ○ Capsule
- ○ Cuillère à soupe
- ○ Cuillère à thé
- ○ Autre

Si vous avez choisi "*Autre*", veuillez **préciser**:

14. Inscrivez **la dose** de ce troisième supplément selon l’unité de mesure choisie à la question précédente.
S'il s'agit d'une multivitamine, inscrivez le nombre de comprimés/capsules.

15. À quelle **fréquence** prenez-vous cette dose?

- ○ 1 fois par jour
- ○ 2 fois par jour
- ○ 3 fois par jour
- ○ Au besoin
- ○ Autre

Précisez :

16. Dans le dernier mois, avez-vous pris un **autre** supplément alimentaire (vitamine/minéraux)?
*Attention: Considérez **aussi** les suppléments alimentaires vendus sous la forme d'huiles (ex. huile de foie de morue).*

- ○ Oui
- ○ Non

17. Inscrivez le nom et le numéro d'identification de ce quatrième supplément.

Nom du supplément [ ]

Numéro d'identification du supplément (DIN) ⓘ [ ]

18. Avec quelle **unité de mesure** souhaitez-vous rapporter la dose de ce quatrième supplément?

- ○ ml
- ○ Goutte
- ○ Comprimé
- ○ Capsule
- ○ Cuillère à soupe
- ○ Cuillère à thé
- ○ Autre

Si vous avez choisi "*Autre*", veuillez **préciser**: [ ]

19. Inscrivez **la dose** de ce quatrième supplément selon l'unité de mesure choisie à la question précédente.
S'il s'agit d'une multivitamine, inscrivez le nombre de comprimés/capsules.

[ ] ⓘ

20. À quelle **fréquence** prenez-vous cette dose?

- ○ 1 fois par jour
- ○ 2 fois par jour
- ○ 3 fois par jour
- ○ Au besoin
- ○ Autre

Précisez : ____________________

21. Dans le dernier mois, avez-vous pris un **autre** supplément alimentaire (vitamine/minéraux)?
*Attention: Considérez **aussi** les suppléments alimentaires vendus sous la forme d'huiles (ex. huile de foie de morue).*

- ○ Oui
- ○ Non

22. Inscrivez le nom et le numéro d'identification de ce cinquième supplément.

Nom du supplément ____________________

Numéro d'identification du supplément (DIN) ⓘ __________

23. Avec quelle **unité de mesure** souhaitez-vous rapporter la dose de ce cinquième supplément?

- ○ ml
- ○ Goutte
- ○ Comprimé
- ○ Capsule
- ○ Cuillère à soupe
- ○ Cuillère à thé
- ○ Autre

Si vous avez choisi "*Autre*", veuillez **préciser**: ____________________

24. Inscrivez **la dose** de ce cinquième supplément selon l'unité de mesure choisie à la question précédente.
S'il s'agit d'une multivitamine, inscrivez le nombre de *<u>comprimés/capsules</u>*.

25. À quelle **fréquence** prenez-vous cette dose?

- ○ 1 fois par jour
- ○ 2 fois par jour
- ○ 3 fois par jour
- ○ Au besoin
- ○ Autre

Précisez :

26. Dans le dernier mois, avez-vous pris un **autre** supplément alimentaire (vitamine/minéraux)?
Attention: Considérez ***aussi*** *les suppléments alimentaires vendus sous la forme d'huiles (ex. huile de foie de morue).*

- ○ Oui
- ○ Non

27. Inscrivez le nom et le numéro d'identification de ce sixième supplément.

Nom du supplément

Numéro d'identification du supplément (DIN)

28.
Avec quelle **unité de mesure** souhaitez-vous rapporter la dose de ce sixième supplément?

- ○ ml
- ○ Goutte
- ○ Comprimé
- ○ Capsule
- ○ Cuillère à soupe
- ○ Cuillère à thé
- ○ Autre

Si vous avez choisi "*Autre*", veuillez **préciser**: ______

29.
Inscrivez **la dose** de ce sixième supplément selon l'unité de mesure choisie à la question précédente.
S'il s'agit d'une multivitamine, inscrivez le nombre de __comprimés/capsules__.

______ ⓘ

30.
À quelle **fréquence** prenez-vous cette dose?

- ○ 1 fois par jour
- ○ 2 fois par jour
- ○ 3 fois par jour
- ○ Au besoin
- ○ Autre

Précisez : ______

31.
Dans le dernier mois, avez-vous pris un **autre** supplément alimentaire (vitamine/minéraux)?
*Attention: Considérez **aussi** les suppléments alimentaires vendus sous la forme d'huiles (ex. huile de foie de morue).*

- ○ Oui
- ○ Non

32. Inscrivez le nom et le numéro d'identification de ce septième supplément.

Nom du supplément ______

Numéro d'identification du supplément (DIN) ⓘ ______

33. Avec quelle **unité de mesure** souhaitez-vous rapporter la dose de ce septième supplément?

- ○ ml
- ○ Goutte
- ○ Comprimé
- ○ Capsule
- ○ Cuillère à soupe
- ○ Cuillère à thé
- ○ Autre

Si vous avez choisi "*Autre*", veuillez **préciser**: ______

34. Inscrivez **la dose** de ce septième supplément selon l'unité de mesure choisie à la question précédente.
S'il s'agit d'une multivitamine, inscrivez le nombre de ***comprimés/capsules***.

______ ⓘ

35. À quelle **fréquence** prenez-vous cette dose?

- ○ 1 fois par jour
- ○ 2 fois par jour
- ○ 3 fois par jour
- ○ Au besoin
- ○ Autre

Précisez : ______

36. Dans le dernier mois, avez-vous pris un **autre** supplément alimentaire (vitamine/minéraux)?
*Attention: Considérez **aussi** les suppléments alimentaires vendus sous la forme d'huiles (ex. huile de foie de morue).*

- ○ Oui
- ○ Non

37. Inscrivez le nom et le numéro d'identification de ce huitième supplément.

Nom du supplément [ ]

Numéro d'identification du supplément (DIN) ⓘ [ ]

38. Avec quelle **unité de mesure** souhaitez-vous rapporter la dose de ce huitième supplément?

- ○ ml
- ○ Goutte
- ○ Comprimé
- ○ Capsule
- ○ Cuillère à soupe
- ○ Cuillère à thé
- ○ Autre

Si vous avez choisi "*Autre*", veuillez **préciser**: [ ]

39. Inscrivez **la dose** de ce huitième supplément selon l'unité de mesure choisie à la question précédente.
S'il s'agit d'une multivitamine, inscrivez le nombre de <u>comprimés/capsules</u>.

[ ] ⓘ

40. À quelle **fréquence** prenez-vous cette dose?

- ○ 1 fois par jour
- ○ 2 fois par jour
- ○ 3 fois par jour
- ○ Au besoin
- ○ Autre

Précisez : ____________

41. Dans le dernier mois, avez-vous pris un **autre** supplément alimentaire (vitamine/minéraux)?
*Attention: Considérez **aussi** les suppléments alimentaires vendus sous la forme d'huiles (ex. huile de foie de morue).*

- ○ Oui
- ○ Non

42. Inscrivez le nom et le numéro d'identification de ce neuvième supplément.

Nom du supplément ____________

Numéro d'identification du supplément (DIN) ⓘ ____________

43. Avec quelle **unité de mesure** souhaitez-vous rapporter la dose de ce neuvième supplément?

- ○ ml
- ○ Goutte
- ○ Comprimé
- ○ Capsule
- ○ Cuillère à soupe
- ○ Cuillère à thé
- ○ Autre

Si vous avez choisi "*Autre*", veuillez **préciser**: ____________

44. Inscrivez **la dose** de ce neuvième supplément selon l'unité de mesure choisie à la question précédente.
S'il s'agit d'une multivitamine, inscrivez le nombre de *<u>comprimés/capsules</u>*.

______ ⓘ

45. À quelle **fréquence** prenez-vous cette dose?

- ○ 1 fois par jour
- ○ 2 fois par jour
- ○ 3 fois par jour
- ○ Au besoin
- ○ Autre

Précisez : ______

46. Dans le dernier mois, avez-vous pris un **autre** supplément alimentaire (vitamine/minéraux)?
*Attention: Considérez **aussi** les suppléments alimentaires vendus sous la forme d'huiles (ex. huile de foie de morue).*

- ○ Oui
- ○ Non

47. Inscrivez le nom et le numéro d'identification de ce dixième supplément.

Nom du supplément ______

Numéro d'identification du supplément (DIN) ⓘ ______

48.
Avec quelle **unité de mesure** souhaitez-vous rapporter la dose de ce dixième supplément?

- ○ ml
- ○ Goutte
- ○ Comprimé
- ○ Capsule
- ○ Cuillère à soupe
- ○ Cuillère à thé
- ○ Autre

Si vous avez choisi "*Autre*", veuillez **préciser**: ______________________

49.
Inscrivez **la dose** de ce dixième supplément selon l'unité de mesure choisie à la question précédente.
S'il s'agit d'une multivitamine, inscrivez le nombre de comprimés/capsules.

__________ ⓘ

50.
À quelle **fréquence** prenez-vous cette dose?

- ○ 1 fois par jour
- ○ 2 fois par jour
- ○ 3 fois par jour
- ○ Au besoin
- ○ Autre

Précisez : ______________________

Supplementary materials 2-D. Questionnaire on supplement use and other variables

QUESTIONNAIRE SUR LES SUPPLÉMENTS ALIMENTAIRES ET AUTRES VARIABLES DURANT LA GROSSESSE

Suppléments alimentaires

1.
Dans le dernier mois, avez-vous pris un supplément alimentaire (vitamine/minéraux)?
*Attention: Considérez **aussi** les suppléments alimentaires vendus sous la forme d'huiles (ex. huile de foie de morue).*

○ Oui

○ Non

2.
Inscrivez le nom et le numéro d'identification de ce supplément.

Nom du supplément ____________

Numéro d'identification du supplément (DIN) ⓘ ____________

3.
Avec quelle **unité de mesure** souhaitez-vous rapporter la dose de ce supplément?

○ ml

○ Goutte

○ Comprimé

○ Capsule

○ Cuillère à soupe

○ Cuillère à thé

○ Autre

Si vous avez choisi "*Autre*", veuillez **préciser**: ____________

4.
Inscrivez **la dose** de votre supplément selon l'unité de mesure choisie à la question précédente.
S'il s'agit d'une multivitamine, inscrivez le nombre de comprimés/capsules.

5.
À quelle **fréquence** prenez-vous cette dose?

- ○ 1 fois par jour
- ○ 2 fois par jour
- ○ 3 fois par jour
- ○ Au besoin
- ○ Autre

Précisez :

6.
Dans le dernier mois, avez-vous pris un **autre** supplément alimentaire (vitamine/minéraux)?
*Attention: Considérez **aussi** les suppléments alimentaires vendus sous la forme d'huiles (ex. huile de foie de morue).*

- ○ Oui
- ○ Non

7.
Inscrivez le nom et le numéro d'identification de ce deuxième supplément.

Nom du supplément

Numéro d'identification du supplément (DIN)

8. Avec quelle **unité de mesure** souhaitez-vous rapporter la dose de ce deuxième supplément?

- ○ ml
- ○ Goutte
- ○ Comprimé
- ○ Capsule
- ○ Cuillère à soupe
- ○ Cuillère à thé
- ○ Autre

Si vous avez choisi "*Autre*", veuillez **préciser**: ______

9. Inscrivez **la dose** de ce deuxième supplément selon l'unité de mesure choisie à la question précédente.
S'il s'agit d'une multivitamine, inscrivez le nombre de comprimés/capsules.

______ ⓘ

10. À quelle **fréquence** prenez-vous cette dose?

- ○ 1 fois par jour
- ○ 2 fois par jour
- ○ 3 fois par jour
- ○ Au besoin
- ○ Autre

Précisez : ______

11. Dans le dernier mois, avez-vous pris un **autre** supplément alimentaire (vitamine/minéraux)?
*Attention: Considérez **aussi** les suppléments alimentaires vendus sous la forme d'huiles (ex. huile de foie de morue).*

- ○ Oui
- ○ Non

12. Inscrivez le nom et le numéro d'identification de ce troisième supplément.

Nom du supplément

Numéro d'identification du supplément (DIN)

13. Avec quelle **unité de mesure** souhaitez-vous rapporter la dose de ce troisième supplément?

- ○ ml
- ○ Goutte
- ○ Comprimé
- ○ Capsule
- ○ Cuillère à soupe
- ○ Cuillère à thé
- ○ Autre

Si vous avez choisi "*Autre*", veuillez **préciser**:

14. Inscrivez **la dose** de ce troisième supplément selon l'unité de mesure choisie à la question précédente.
S'il s'agit d'une multivitamine, inscrivez le nombre de comprimés/capsules.

15. À quelle **fréquence** prenez-vous cette dose?

- ○ 1 fois par jour
- ○ 2 fois par jour
- ○ 3 fois par jour
- ○ Au besoin
- ○ Autre

Précisez :

16.

Dans le dernier mois, avez-vous pris un **autre** supplément alimentaire (vitamine/minéraux)?
*Attention: Considérez **aussi** les suppléments alimentaires vendus sous la forme d'huiles (ex. huile de foie de morue).*

- ○ Oui
- ○ Non

17.

Inscrivez le nom et le numéro d'identification de ce quatrième supplément.

Nom du supplément [ ]

Numéro d'identification du supplément (DIN) ⓘ [ ]

18.

Avec quelle **unité de mesure** souhaitez-vous rapporter la dose de ce quatrième supplément?

- ○ ml
- ○ Goutte
- ○ Comprimé
- ○ Capsule
- ○ Cuillère à soupe
- ○ Cuillère à thé
- ○ Autre

Si vous avez choisi "*Autre*", veuillez **préciser**: [ ]

19.

Inscrivez **la dose** de ce quatrième supplément selon l'unité de mesure choisie à la question précédente.
S'il s'agit d'une multivitamine, inscrivez le nombre de <u>comprimés/capsules</u>.

[ ] ⓘ

20. À quelle **fréquence** prenez-vous cette dose?

- ○ 1 fois par jour
- ○ 2 fois par jour
- ○ 3 fois par jour
- ○ Au besoin
- ○ Autre

Précisez : ______

21. Dans le dernier mois, avez-vous pris un **autre** supplément alimentaire (vitamine/minéraux)?
*Attention: Considérez **aussi** les suppléments alimentaires vendus sous la forme d'huiles (ex. huile de foie de morue).*

- ○ Oui
- ○ Non

22. Inscrivez le nom et le numéro d'identification de ce cinquième supplément.

Nom du supplément ______

Numéro d'identification du supplément (DIN) ⓘ ______

23. Avec quelle **unité de mesure** souhaitez-vous rapporter la dose de ce cinquième supplément?

- ○ ml
- ○ Goutte
- ○ Comprimé
- ○ Capsule
- ○ Cuillère à soupe
- ○ Cuillère à thé
- ○ Autre

Si vous avez choisi "*Autre*", veuillez **préciser**: ______

24.
Inscrivez **la dose** de ce cinquième supplément selon l'unité de mesure choisie à la question précédente.
S'il s'agit d'une multivitamine, inscrivez le nombre de comprimés/capsules.

25.
À quelle **fréquence** prenez-vous cette dose?

- ○ 1 fois par jour
- ○ 2 fois par jour
- ○ 3 fois par jour
- ○ Au besoin
- ○ Autre

Précisez :

26.
Dans le dernier mois, avez-vous pris un **autre** supplément alimentaire (vitamine/minéraux)?
*Attention: Considérez **aussi** les suppléments alimentaires vendus sous la forme d'huiles (ex. huile de foie de morue).*

- ○ Oui
- ○ Non

27.
Inscrivez le nom et le numéro d'identification de ce sixième supplément.

Nom du supplément

Numéro d'identification du supplément (DIN)

28.
Avec quelle **unité de mesure** souhaitez-vous rapporter la dose de ce sixième supplément?

- ○ ml
- ○ Goutte
- ○ Comprimé
- ○ Capsule
- ○ Cuillère à soupe
- ○ Cuillère à thé
- ○ Autre

Si vous avez choisi "*Autre*", veuillez **préciser**: ______

29.
Inscrivez **la dose** de ce sixième supplément selon l'unité de mesure choisie à la question précédente.
S'il s'agit d'une multivitamine, inscrivez le nombre de comprimés/capsules.

______ ⓘ

30.
À quelle **fréquence** prenez-vous cette dose?

- ○ 1 fois par jour
- ○ 2 fois par jour
- ○ 3 fois par jour
- ○ Au besoin
- ○ Autre

Précisez : ______

31.
Dans le dernier mois, avez-vous pris un **autre** supplément alimentaire (vitamine/minéraux)?
*Attention: Considérez **aussi** les suppléments alimentaires vendus sous la forme d'huiles (ex. huile de foie de morue).*

- ○ Oui
- ○ Non

32. Inscrivez le nom et le numéro d'identification de ce septième supplément.

Nom du supplément [ ]

Numéro d'identification du supplément (DIN) ⓘ [ ]

33. Avec quelle **unité de mesure** souhaitez-vous rapporter la dose de ce septième supplément?

- ○ ml
- ○ Goutte
- ○ Comprimé
- ○ Capsule
- ○ Cuillère à soupe
- ○ Cuillère à thé
- ○ Autre

Si vous avez choisi "*Autre*", veuillez **préciser**: [ ]

34. Inscrivez **la dose** de ce septième supplément selon l'unité de mesure choisie à la question précédente.
S'il s'agit d'une multivitamine, inscrivez le nombre de comprimés/capsules.

[ ] ⓘ

35. À quelle **fréquence** prenez-vous cette dose?

- ○ 1 fois par jour
- ○ 2 fois par jour
- ○ 3 fois par jour
- ○ Au besoin
- ○ Autre

Précisez : [ ]

36.
Dans le dernier mois, avez-vous pris un **autre** supplément alimentaire (vitamine/minéraux)?
*Attention: Considérez **aussi** les suppléments alimentaires vendus sous la forme d'huiles (ex. huile de foie de morue).*

- ○ Oui
- ○ Non

37.
Inscrivez le nom et le numéro d'identification de ce huitième supplément.

Nom du supplément [ ]

Numéro d'identification du supplément (DIN) ⓘ [ ]

38.
Avec quelle **unité de mesure** souhaitez-vous rapporter la dose de ce huitième supplément?

- ○ ml
- ○ Goutte
- ○ Comprimé
- ○ Capsule
- ○ Cuillère à soupe
- ○ Cuillère à thé
- ○ Autre

Si vous avez choisi "*Autre*", veuillez **préciser**: [ ]

39.
Inscrivez **la dose** de ce huitième supplément selon l'unité de mesure choisie à la question précédente.
S'il s'agit d'une multivitamine, inscrivez le nombre de comprimés/capsules.

[ ] ⓘ

40. À quelle **fréquence** prenez-vous cette dose?

- ○ 1 fois par jour
- ○ 2 fois par jour
- ○ 3 fois par jour
- ○ Au besoin
- ○ Autre

Précisez : ______

41. Dans le dernier mois, avez-vous pris un **autre** supplément alimentaire (vitamine/minéraux)?
*Attention: Considérez **aussi** les suppléments alimentaires vendus sous la forme d'huiles (ex. huile de foie de morue).*

- ○ Oui
- ○ Non

42. Inscrivez le nom et le numéro d'identification de ce neuvième supplément.

Nom du supplément ______

Numéro d'identification du supplément (DIN) ⓘ ______

43. Avec quelle **unité de mesure** souhaitez-vous rapporter la dose de ce neuvième supplément?

- ○ ml
- ○ Goutte
- ○ Comprimé
- ○ Capsule
- ○ Cuillère à soupe
- ○ Cuillère à thé
- ○ Autre

Si vous avez choisi "*Autre*", veuillez **préciser**: ______

44. Inscrivez **la dose** de ce neuvième supplément selon l'unité de mesure choisie à la question précédente.
S'il s'agit d'une multivitamine, inscrivez le nombre de comprimés/capsules.

45. À quelle **fréquence** prenez-vous cette dose?

- ○ 1 fois par jour
- ○ 2 fois par jour
- ○ 3 fois par jour
- ○ Au besoin
- ○ Autre

Précisez :

46. Dans le dernier mois, avez-vous pris un **autre** supplément alimentaire (vitamine/minéraux)?
*Attention: Considérez **aussi** les suppléments alimentaires vendus sous la forme d'huiles (ex. huile de foie de morue).*

- ○ Oui
- ○ Non

47. Inscrivez le nom et le numéro d'identification de ce dixième supplément.

Nom du supplément

Numéro d'identification du supplément (DIN)

48.
Avec quelle **unité de mesure** souhaitez-vous rapporter la dose de ce dixième supplément?

- ○ ml
- ○ Goutte
- ○ Comprimé
- ○ Capsule
- ○ Cuillère à soupe
- ○ Cuillère à thé
- ○ Autre

Si vous avez choisi "*Autre*", veuillez **préciser**: ______

49.
Inscrivez **la dose** de ce dixième supplément selon l'unité de mesure choisie à la question précédente.
S'il s'agit d'une multivitamine, inscrivez le nombre de comprimés/capsules.

______ ⓘ

50.
À quelle **fréquence** prenez-vous cette dose?

- ○ 1 fois par jour
- ○ 2 fois par jour
- ○ 3 fois par jour
- ○ Au besoin
- ○ Autre

Précisez : ______

Nausées

51.
Dans le dernier mois, avez-vous eu des **nausées**?

- ○ Oui
- ○ Non

52.
Dans le dernier mois, à quelle **fréquence** avez-vous eu des **nausées**?

- ○ 2 fois par jour ou plus
- ○ 1 fois par jour
- ○ 3 à 6 fois par semaine
- ○ 1 à 2 fois par semaine
- ○ Occasionnellement
- ○ Rarement

Vomissements

53.
Dans le dernier mois, avez-vous eu des **vomissements**?

- ○ Oui
- ○ Non

54. Dans le dernier mois, à quelle **fréquence** avez-vous eu des **vomissements**?

- ○ 2 fois par jour ou plus
- ○ 1 fois par jour
- ○ 3 à 6 fois par semaine
- ○ 1 à 2 fois par semaine
- ○ Occasionnellement
- ○ Rarement

Préférences alimentaires

55. Dans le dernier mois, avez-vous ressenti des **désirs/envies intenses** pour certains aliments?

- ○ Oui
- ○ Non

56. Listez ces aliments de façon concise. N'inscrivez qu'un seul aliment par case et utilisez autant de cases que nécessaire.

57.
Dans le dernier mois, avez-vous mangé ces aliments en réponse à ces désirs/envies intenses?

- Oui
- Non

Aversions alimentaires

58.
Dans le dernier mois, avez-vous ressenti un **dédain/une aversion intense** pour certains aliments?

- Oui
- Non

59.
Listez ces aliments de façon concise. N'inscrivez qu'un seul aliment par case et utilisez autant de cases que nécessaire.

Supplementary materials 2-E. International Physical Activity Questionnaire – Short Form (IPAQ-SF)

IPAQ - QUESTIONNAIRE AUTO-ADMINISTRÉ SUR L'ACTIVITÉ PHYSIQUE

Toutes les réponses sont importantes pour les chercheurs. Toutefois, vous pouvez choisir d'y répondre complètement ou partiellement. Notez également qu'à tout moment vous pouvez vous retirer du projet.

Instructions

Nous sommes intéressés à connaître les types d'activités physiques que les gens font dans leur vie quotidienne. Les questions concerneront le temps que vous avez passé à être physiquement actif(ve) au cours des **7 derniers jours**. Veuillez répondre à chaque question même si vous ne vous considérez pas comme une personne active. Pensez aux activités que vous faites au travail, en travaillant dans la maison et dans le jardin, en vous rendant d'un lieu à l'autre et, à titre de loisir, d'exercice ou de sport.

Veuillez penser d'abord à toutes les activités d'intensité **élevée** que vous avez faites au cours des **7 derniers jours**. Les activités d'intensité **élevée** sont des activités qui exigent un grand effort physique et qui vous font souffler beaucoup plus fort que d'habitude. Pensez *seulement* aux fois que vous avez fait ces activités pendant au moins 10 minutes à chaque fois.

1. Au cours des **7 derniers jours**, combien de jours avez-vous fait des activités d'intensité **élevée** comme soulever quelque chose de lourd, faire des exercices d'aérobie ou se déplacer rapidement à bicyclette?

[ ] jour(s) par semaine

○ Aucune activité physique d'intensité élevée

2. Ces jours-là, combien de temps avez-vous passé généralement **par jour** à faire des activités d'intensité **élevée**? ⓘ

Nombre de minute(s) ou d'heure(s) en moyenne par jour
00 ⌄ 00 ⌄ (HH:MM)

○ Je ne sais pas

Pensez à toutes les activités d'intensité **moyenne** que vous avez faites au cours des **7 derniers jours**. Les activités d'intensité **moyenne** sont des activités qui exigent un effort physique moyen et qui vous font souffler un peu plus fort que d'habitude. Pensez *seulement* aux fois que vous avez fait ces activités pendant au moins 10 minutes à chaque fois.

3. Au cours des **7 derniers jours**, combien de jours avez-vous fait des activités d'intensité **moyenne** comme transporter quelque chose de léger, se déplacer à bicyclette à une vitesse régulière ou à jouer au tennis en double? Elles n'incluent pas la marche.

[ ] jour(s) par semaine

○ Aucune activité d'intensité moyenne

4. Ces jours-là, combien de temps avez-vous passé généralement **par jour** à faire des activités d'intensité **moyenne**? ⓘ

Nombres de minute(s) ou d'heure(s) en moyenne par jour
00 ⌄ 00 ⌄ (HH:MM)

○ Je ne sais pas

Veuillez penser maintenant au temps que vous avez passé à **marcher** pendant les **7 derniers jours**. Cela comprend la marche au travail et à la maison, la marche en tant que mode de transport d'un lieu à un autre et la marche à titre de loisir, d'exercice ou de sport.

5. Combien de jours, au cours des **7 derniers jours**, avez-vous **marché** pendant au moins 10 minutes à chaque fois?

[ ] jour(s) par semaine

○ Aucun

6. Ces jours-là, combien de temps avez-vous passé généralement par jour à **marcher**? ⓘ

Nombres de minute(s) ou d'heure(s) en moyenne par jour
00 ⌄ 00 ⌄ (HH:MM)

Maintenant, veuillez penser au temps que vous avez passé **assis(e)** pendant les jours de la semaine, au cours des **7 derniers jours**.

Veuillez inclure le temps passé ainsi au travail, à la maison, à faire des travaux de cours et pendant vos loisirs. Cela peut aussi inclure le temps que vous passé assis(e) à un bureau, à rendre visite à des amis, à lire, à voyager en autobus ou le temps passé assis(e) ou allongé(e) à regarder la télévision.

7. Pendant les **7 derniers jours**, combien de temps avez-vous passé **assis(e)** par jour de la semaine?

Nombres de minute(s) ou d'heure(s) en moyenne par jour
00 ⌄ 00 ⌄ (HH:MM)

○ Je ne sais pas

Supplementary materials 2-F. Pregnancy Physical Activity Questionnaire (PPAQ)

ACTIVITÉ PHYSIQUE DERNIER MOIS DE GROSSESSE

Il est très important que vous répondiez honnêtement aux questions. Il n'y a pas de bonne ou de mauvaise réponse. Nous voulons seulement connaître les activités que vous réalisées durant votre grossesse. Dans le DERNIER MOIS, quand vous N'ÉTIEZ PAS au travail, combien de temps passiez-vous généralement à :

1. Préparer les repas (cuisiner, mettre la table, laver la vaisselle).

○ Jamais ○ Moins de ½h / jour ○ ½h à presque 1h / jour ○ 1h à presque 2h / jour ○ 2h à presque 3h / jour ○ 3h ou plus / jour

2. Habiller, laver et nourrir les enfants en étant ASSISE.

○ Jamais ○ Moins de ½h / jour ○ ½h à presque 1h / jour ○ 1h à presque 2h / jour ○ 2h à presque 3h / jour ○ 3h ou plus / jour

3. Habiller, laver et nourrir les enfants en étant DEBOUT.

○ Jamais ○ Moins de ½h / jour ○ ½h à presque 1h / jour ○ 1h à presque 2h / jour ○ 2h à presque 3h / jour ○ 3h ou plus / jour

4. Jouer avec les enfants en étant ASSISE ou DEBOUT.

○ Jamais ○ Moins de ½h / jour ○ ½h à presque 1h / jour ○ 1h à presque 2h / jour ○ 2h à presque 3h / jour ○ 3h ou plus / jour

5. Jouer avec les enfants en MARCHANT ou en COURANT.

○ Jamais ○ Moins de ½h / jour ○ ½h à presque 1h / jour ○ 1h à presque 2h / jour ○ 2h à presque 3h / jour ○ 3h ou plus / jour

6. Porter des enfants (dans les bras, porte-bébé, sur le dos, etc.)

○ Jamais ○ Moins de ½h / jour ○ ½h à presque 1h / jour ○ 1h à presque 2h / jour ○ 2h à presque 3h / jour ○ 3h ou plus / jour

7. Prendre soin d'une personne âgée.

○ Jamais ○ Moins de ½h / jour ○ ½h à presque 1h / jour ○ 1h à presque 2h / jour ○ 2h à presque 3h / jour ○ 3h ou plus / jour

8. Vous asseoir pour utiliser un ordinateur ou écrire, lorsque vous n'êtes PAS au travail.

○ Jamais ○ Moins de ½h / jour ○ ½h à presque 1h / jour ○ 1h à presque 2h / jour ○ 2h à presque 3h / jour ○ 3h ou plus / jour

9. Regarder la télévision ou une vidéo.

○ Jamais ○ Moins de ½h / jour ○ ½h à presque 2h / jour ○ 2h à presque 4h / jour ○ 4h à presque 6h / jour ○ 6h ou plus / jour

10. Vous asseoir pour lire, parler, ou téléphoner, lorsque vous n'êtes PAS au travail.

○ Jamais ○ Moins de ½h / jour ○ ½h à presque 2h / jour ○ 2h à presque 4h / jour ○ 4h à presque 6h / jour ○ 6h ou plus / jour

11. Jouer avec des animaux domestiques.

○ Jamais ○ Moins de ½h / jour ○ ½h à presque 1h / jour ○ 1h à presque 2h / jour ○ 2h à presque 3h / jour ○ 3h ou plus / jour

12. Faire les tâches ménagères habituelles (faire les lits, la lessive, repasser, ranger les choses).

○ Jamais ○ Moins de ½h / jour ○ ½h à presque 1h / jour ○ 1h à presque 2h / jour ○ 2h à presque 3h / jour ○ 3h ou plus / jour

13. Magasiner (nourriture, vêtements, autres).

○ Jamais ○ Moins de ½h / jour ○ ½h à presque 1h / jour ○ 1h à presque 2h / jour ○ 2h à presque 3h / jour ○ 3h ou plus / jour

14. Faire le ménage (passer l'aspirateur, la vadrouille, balayer, laver les fenêtres).

○ Jamais ○ Moins de ½h / sem ○ ½h à presque 1h / sem ○ 1h à presque 2h / sem ○ 2h à presque 3h / sem ○ 3h ou plus / sem

15. Tondre la pelouse à l'aide d'un tracteur à pelouse (position assise), déblayer la neige sans la soulever.

○ Jamais ○ Moins de ½h / sem ○ ½h à presque 1h / sem ○ 1h à presque 2h / sem ○ 2h à presque 3h / sem ○ 3h ou plus / sem

16. Tondre la pelouse à l'aide d'une tondeuse à pelouse (position debout), râteler les feuilles, jardiner, pelleter la neige.

○ Jamais ○ Moins de ½h / sem ○ ½h à presque 1h / sem ○ 1h à presque 2h / sem ○ 2h à presque 3h / sem ○ 3h ou plus / sem

SE DÉPLACER D'UN ENDROIT À L'AUTRE... Dans le DERNIER MOIS, combien de temps passiez-vous généralement à :

17. Marcher LENTEMENT pour vous déplacer à un endroit (par exemple: pour prendre l'autobus, aller au travail, rendre visite). PAS POUR LE PLAISIR OU L'EXERCICE.

○ Jamais ○ Moins de ½h / jour ○ ½h à presque 1h / jour ○ 1h à presque 2h / jour ○ 2h à presque 3h / jour ○ 3h ou plus / jour

18. Marcher RAPIDEMENT pour vous déplacer à un endroit (par exemple: pour prendre l'autobus, aller au travail ou à l'école). PAS POUR LE PLAISIR OU L'EXERCICE.

○ Jamais ○ Moins de ½h / jour ○ ½h à presque 1h / jour ○ 1h à presque 2h / jour ○ 2h à presque 3h / jour ○ 3h ou plus / jour

19. Conduire ou prendre place dans une voiture ou un autobus.

○ Jamais ○ Moins de ½h / jour ○ ½h à presque 1h / jour ○ 1h à presque 2h / jour ○ 2h à presque 3h / jour ○ 3h ou plus / jour

POUR LE PLAISIR OU COMME EXERCICE... Dans le DERNIER MOIS, combien de temps passiez-vous généralement à :

20. Marcher LENTEMENT pour le plaisir ou comme exercice.

○ Jamais ○ Moins de ½h / sem ○ ½h à presque 1h / sem ○ 1h à presque 2h / sem ○ 2h à presque 3h / sem ○ 3h ou plus / sem

21. Marcher RAPIDEMENT pour le plaisir ou comme exercice.

○ Jamais ○ Moins de ½h / sem ○ ½h à presque 1h / sem ○ 1h à presque 2h / sem ○ 2h à presque 3h / sem ○ 3h ou plus / sem

22. Marcher RAPIDEMENT en MONTÉE pour le plaisir ou comme exercice.

○ Jamais ○ Moins de ½h / sem ○ ½h à presque 1h / sem ○ 1h à presque 2h / sem ○ 2h à presque 3h / sem ○ 3h ou plus / sem

23. Jogger

○ Jamais ○ Moins de ½h / sem ○ ½h à presque 1h / sem ○ 1h à presque 2h / sem ○ 2h à presque 3h / sem ○ 3h ou plus / sem

24. Suivre des cours d'exercices prénataux.

- ○ Jamais ○ Moins de ½h / sem ○ ½h à presque 1h / sem ○ 1h à presque 2h / sem ○ 2h à presque 3h / sem ○ 3h ou plus / sem

25. Nager

- ○ Jamais ○ Moins de ½h / sem ○ ½h à presque 1h / sem ○ 1h à presque 2h / sem ○ 2h à presque 3h / sem ○ 3h ou plus / sem

26. Danser

- ○ Jamais ○ Moins de ½h / sem ○ ½h à presque 1h / sem ○ 1h à presque 2h / sem ○ 2h à presque 3h / sem ○ 3h ou plus / sem

27. Avez-vous fait une autre activité pour le plaisir ou comme exercice? S'il-vous-plait, nommez-la.

Activité ______________________

- ○ Jamais
- ○ Moins de ½h / semaine
- ○ ½h à presque 1h / semaine
- ○ 1h à presque 2h / semaine
- ○ 2h à presque 3h / semaine
- ○ 3h ou plus / semaine

28. Avez-vous fait une deuxième activité pour le plaisir ou comme exercice? S'il-vous-plait, nommez-la.

Activité ______________________

- ○ Jamais
- ○ Moins de ½h / semaine
- ○ ½h à presque 1h / semaine
- ○ 1h à presque 2h / semaine
- ○ 2h à presque 3h / semaine
- ○ 3h ou plus / semaine

29. Avez-vous fait une troisième activité pour le plaisir ou comme exercice? S'il-vous-plait, nommez-la.

Activité ____________________

- ○ Jamais
- ○ Moins de ½h / semaine
- ○ ½h à presque 1h / semaine
- ○ 1h à presque 2h / semaine
- ○ 2h à presque 3h / semaine
- ○ 3h ou plus / semaine

30. Avez-vous fait une autre activité pour le plaisir ou comme exercice? S'il-vous-plait, nommez-la.

Activité ____________________

- ○ Jamais
- ○ Moins de ½h / semaine
- ○ ½h à presque 1h / semaine
- ○ 1h à presque 2h / semaine
- ○ 2h à presque 3h / semaine
- ○ 3h ou plus / semaine

31. Avez-vous fait une autre activité pour le plaisir ou comme exercice? S'il-vous-plait, nommez-la.

Activité ____________________

- ○ Jamais
- ○ Moins de ½h / semaine
- ○ ½h à presque 1h / semaine
- ○ 1h à presque 2h / semaine
- ○ 2h à presque 3h / semaine
- ○ 3h ou plus / semaine

AU TRAVAIL... S'il vous plaît, complétez la prochaine section si, au cours du mois dernier, vous travaillez avec rémunération, comme bénévole ou si vous étiez étudiante. Si vous étiez au foyer, en retrait préventif à la maison, sans emploi ou inapte au travail, vous n'avez pas besoin de remplir cette dernière section. Dans le DERNIER MOIS, combien de temps passiez-vous généralement à :

32. Être assise pendant le travail ou en classe.

○ Jamais ○ Moins de ½h / jour ○ ½h à presque 2h / jour ○ 2h à presque 4h / jour ○ 4h à presque 6h / jour ○ 6h ou plus / jour

33. Être debout ou marcher LENTEMENT pendant le travail tout en transportant des choses plus lourdes qu'un 4 litres (1 gallon) de lait.

○ Jamais ○ Moins de ½h / jour ○ ½h à presque 2h / jour ○ 2h à presque 4h / jour ○ 4h à presque 6h / jour ○ 6h ou plus / jour

34. Être debout ou marcher LENTEMENT pendant le travail SANS transporter quoi que ce soit.

○ Jamais ○ Moins de ½h / jour ○ ½h à presque 2h / jour ○ 2h à presque 4h / jour ○ 4h à presque 6h / jour ○ 6h ou plus / jour

35. Marcher RAPIDEMENT pendant le travail tout en transportant des choses plus lourdes qu'un 4 litres (1 gallon) de lait.

○ Jamais ○ Moins de ½h / jour ○ ½h à presque 2h / jour ○ 2h à presque 4h / jour ○ 4h à presque 6h / jour ○ 6h ou plus / jour

36. Marcher RAPIDEMENT pendant le travail SANS transporter quoi que ce soit.

○ Jamais ○ Moins de ½h / jour ○ ½h à presque 2h / jour ○ 2h à presque 4h / jour ○ 4h à presque 6h / jour ○ 6h ou plus / jour

24-hour Dietary Recall (R24W)

Context

R24W is an automated, self-administered, web-based 24h dietary recall that collects food intake data with minimal bias.

Description of Respondent Web Site

R24W respondent Web site solicits detailed information about foods and beverages that have been consumed from midnight to midnight the previous day.

The application:

- Provides a mandatory tutorial about 5 minutes at the first connection to provide some instructions and enhance use in low-literacy respondents.
- Is designed as a meal-based approach which means that a meal or a snack must be created before selecting foods (Figure 1);
- Asks questions about meal or snack context (time, location, with whom) and screen-based activities during meals (Figure 2);
- Allows selection of foods and drinks by browsing food categories or by using a search engine. (Figure 3 and Figure 4);
- Contains images to help in estimating portion sizes (Figure 5 to 10),
- Asks questions about potential additions or toppings (e.g. sauces, condiments, seasonings) (Figure 11);
- Provides an overview of eight food categories frequently forgotten at the end of each meal and at the end of the 24HDR (Figure 12);
- Includes a final review of the day's intakes (Figure 13);
- Includes questions about factors that influence nutritional needs (age, gender, pregnancy, breastfeeding, smoking), whether the day's intakes were usual or not, adherence to a dietary regimen, and supplement or natural health product intakes (Figure 14 to 18);
- Does not provide any direct feedback to respondents.

Methods

Foods and Drinks Selection

To find a food or beverage, it is possible to browse an organized food list. This list contains 2,865 items divided into 16 categories and 98 subcategories and includes 687 recipes of mixed dishes including 386 multi-ethnic dishes. It is also possible to use a search tool. Synonyms and trademark names have been associated with food and beverages to increase research efficiency.

Portion Selection

A total of 1491 pictures are used for the selection of portions; each food being illustrated by 0 to 8 pictures. Each picture has a description in unit and / or volume (ml) and / or weight (g). Sometimes, commercial containers appear on the pictures to assist the respondent in identifying one's portion. It is also possible to indicate that the portion consumed was smaller than the first image or bigger than the last image. In this case, a fixed quantity, unknown by the respondent, is assigned. Finally, a drop-down menu makes it possible to divide or multiply the portion shown in the selected picture.

Appendix 1: Screenshots of the Web Application

Figure 1: Selection of meals and snacks

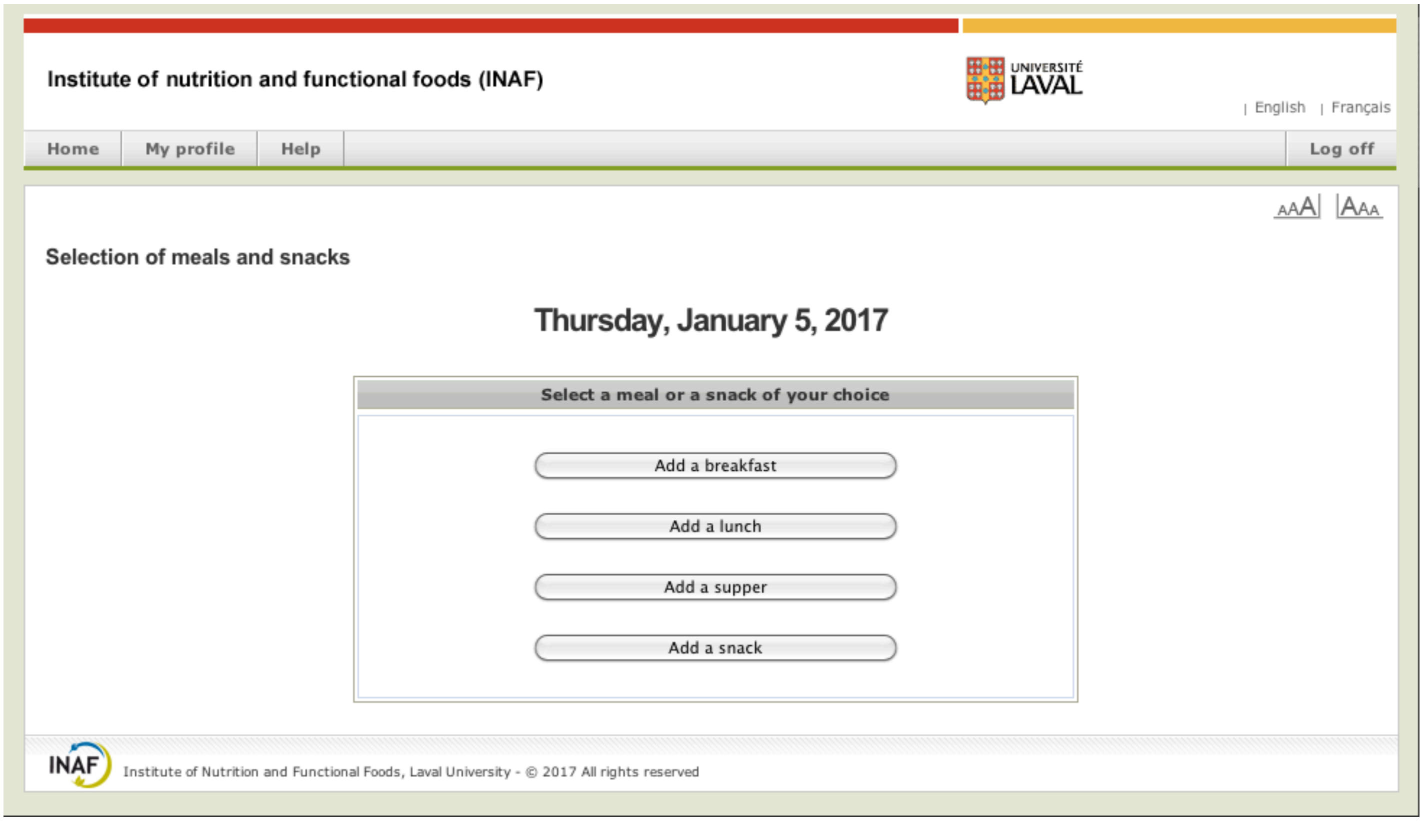

Figure 2: Questions about the context of the meal

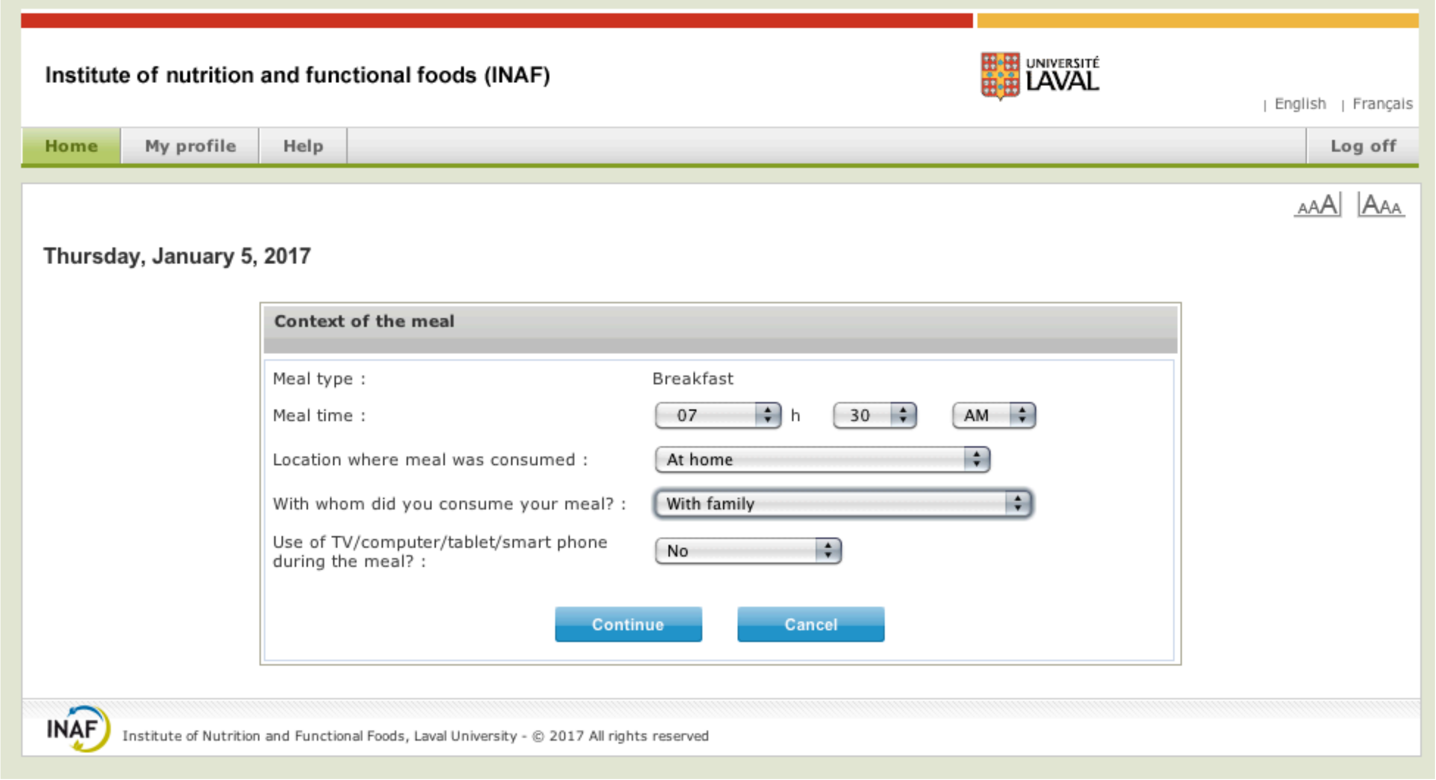

Figure 3: Food and drink selection with structured food list

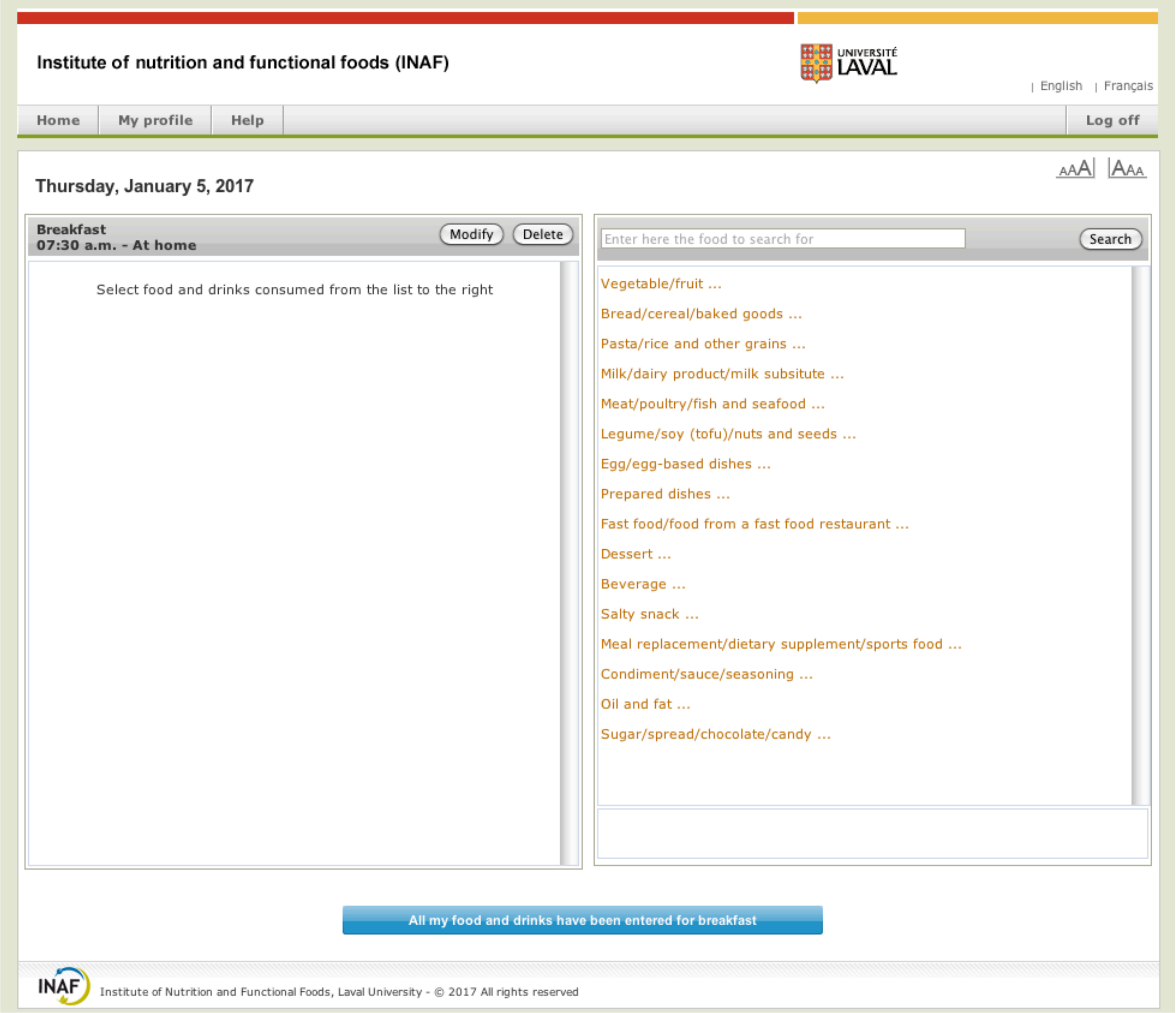

Figure 4: Selection of food and drinks with the search engine

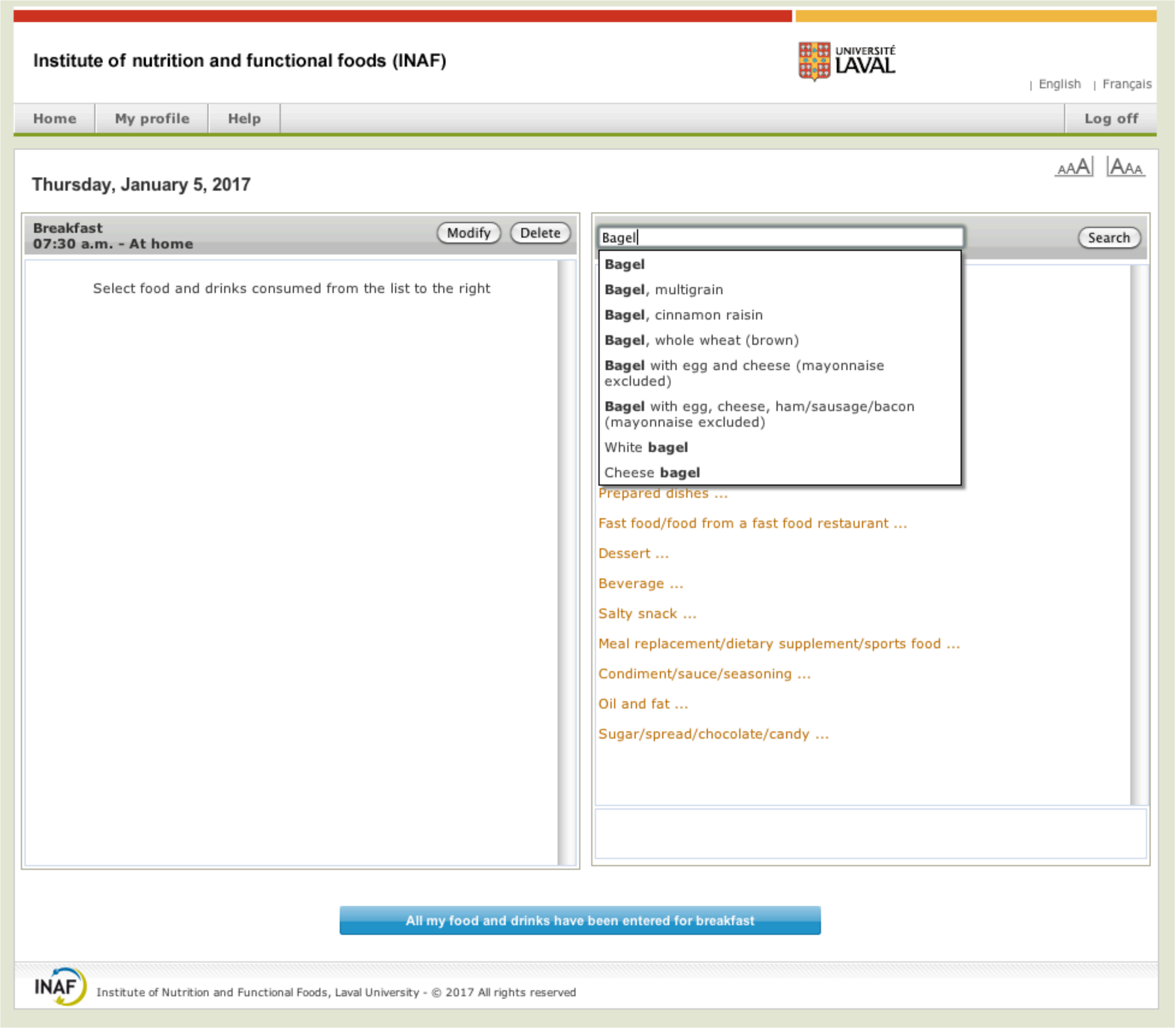

Figure 5: Portion selection with household or commercial containers

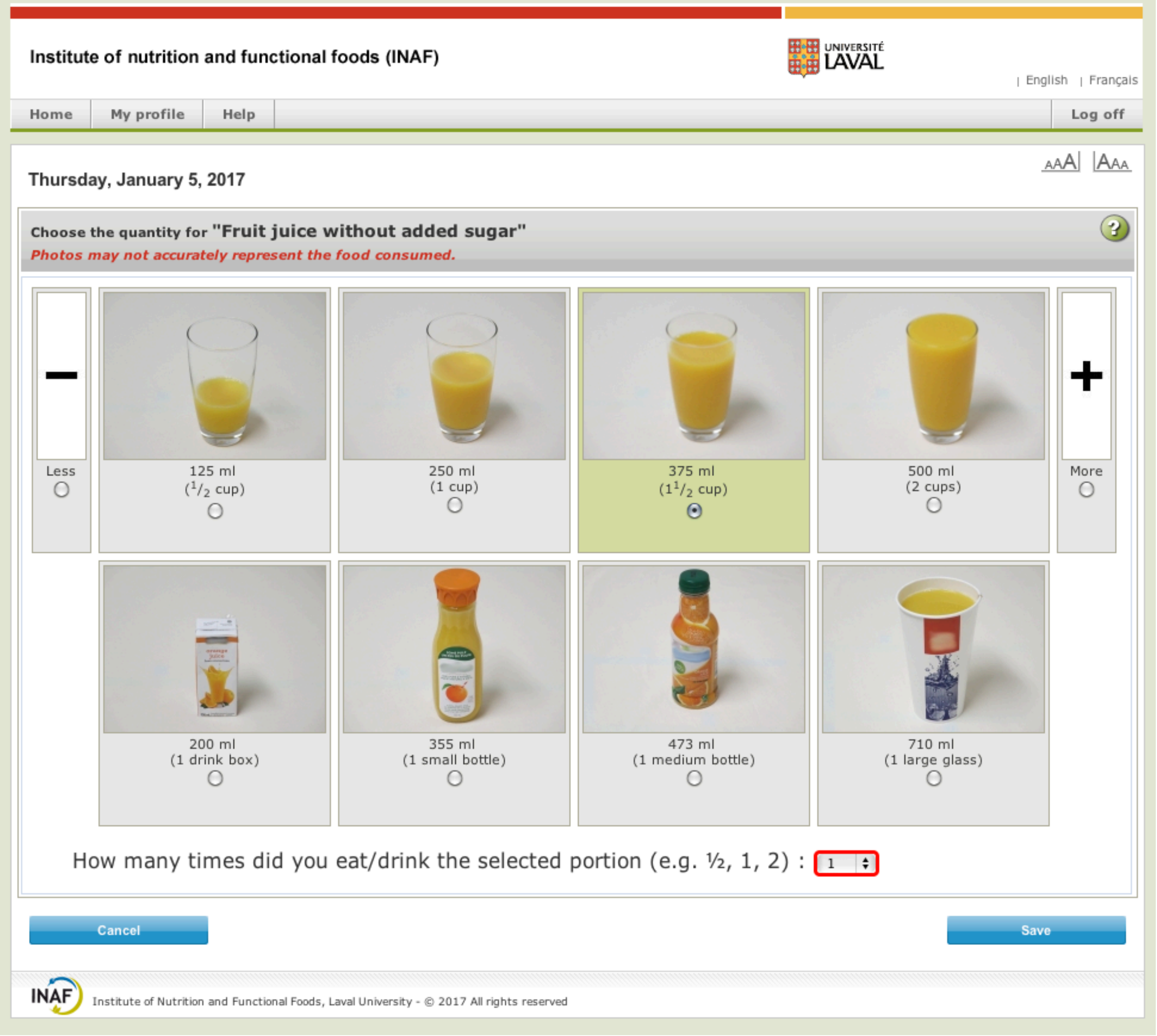

Figure 6: Portion selection with household measures and commercial packings

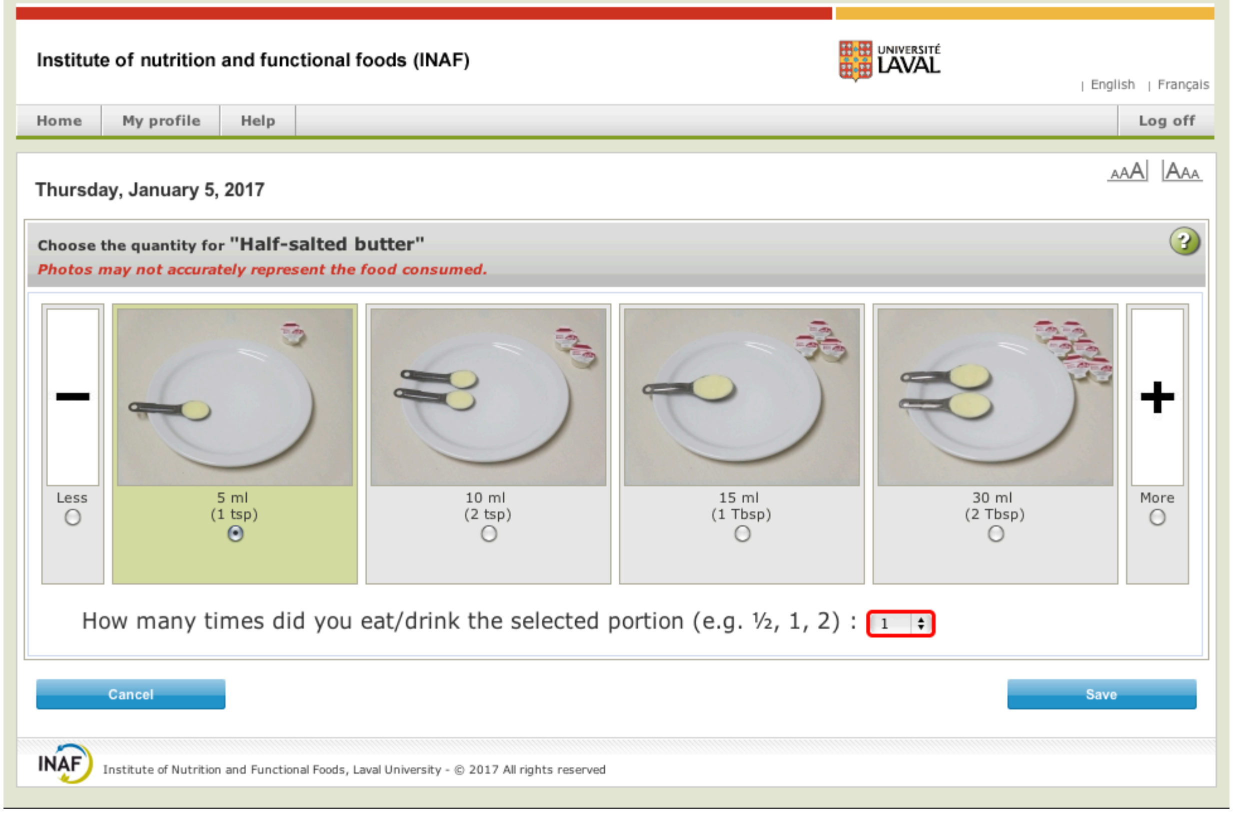

Figure 7: Portion selection with household measures

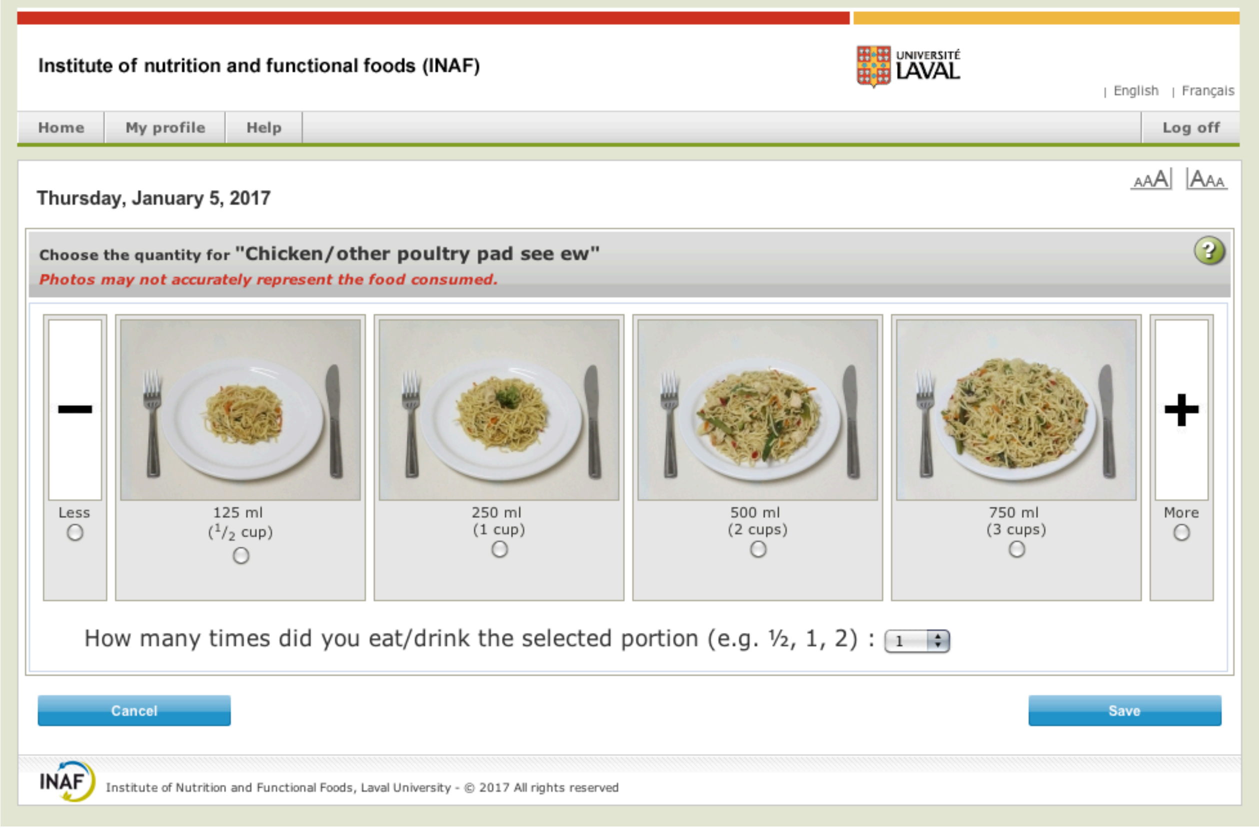

Figure 8: Portion selection with pieces and weights

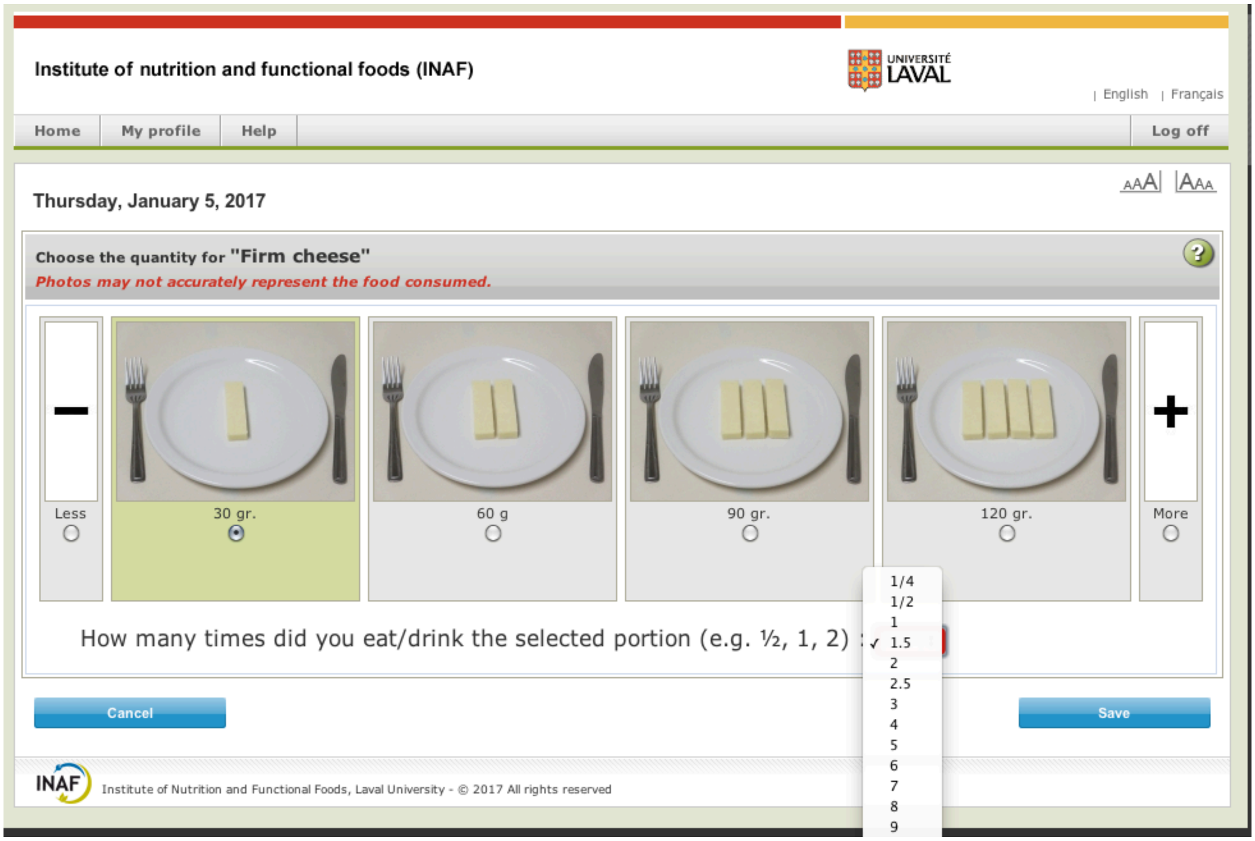

Figure 9: Portion selection with fractions of the whole food

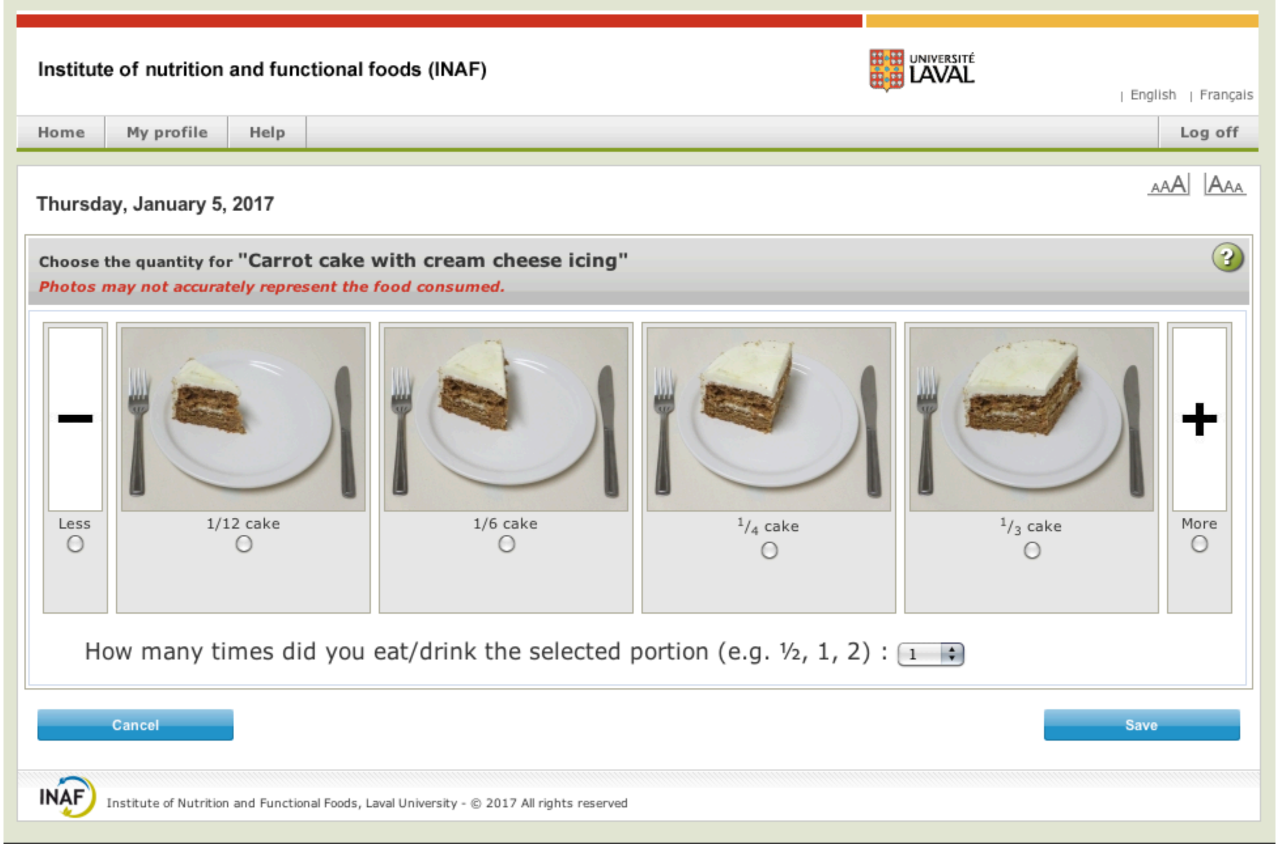

Figure 10: Portion selection with a drop-down menu

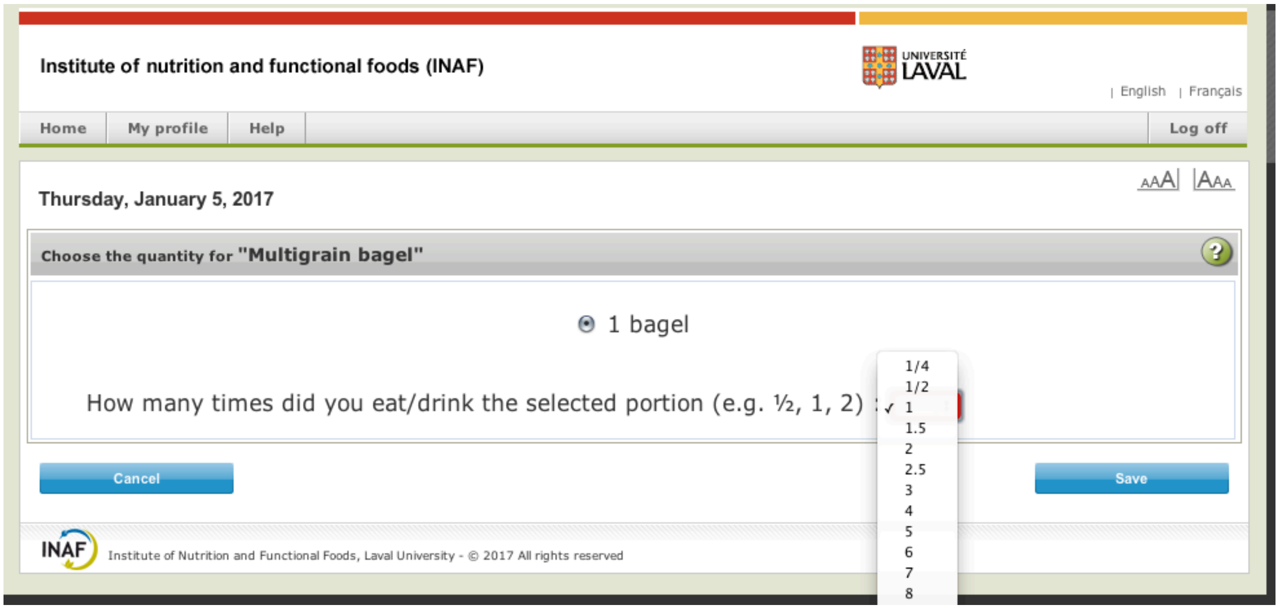

Figure 11: Selection of additions

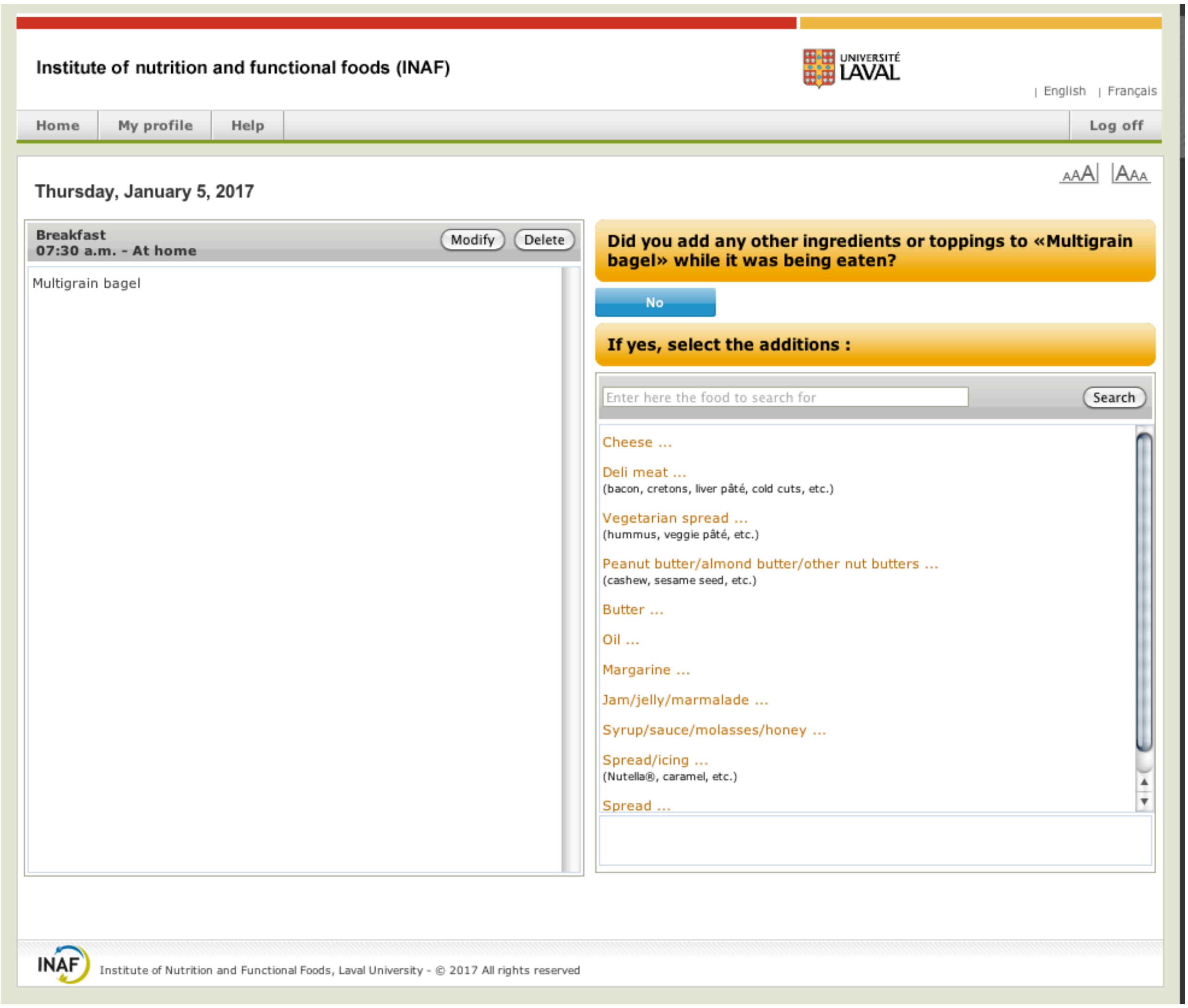

Figure 12: Question about food and drinks that could have been forgotten

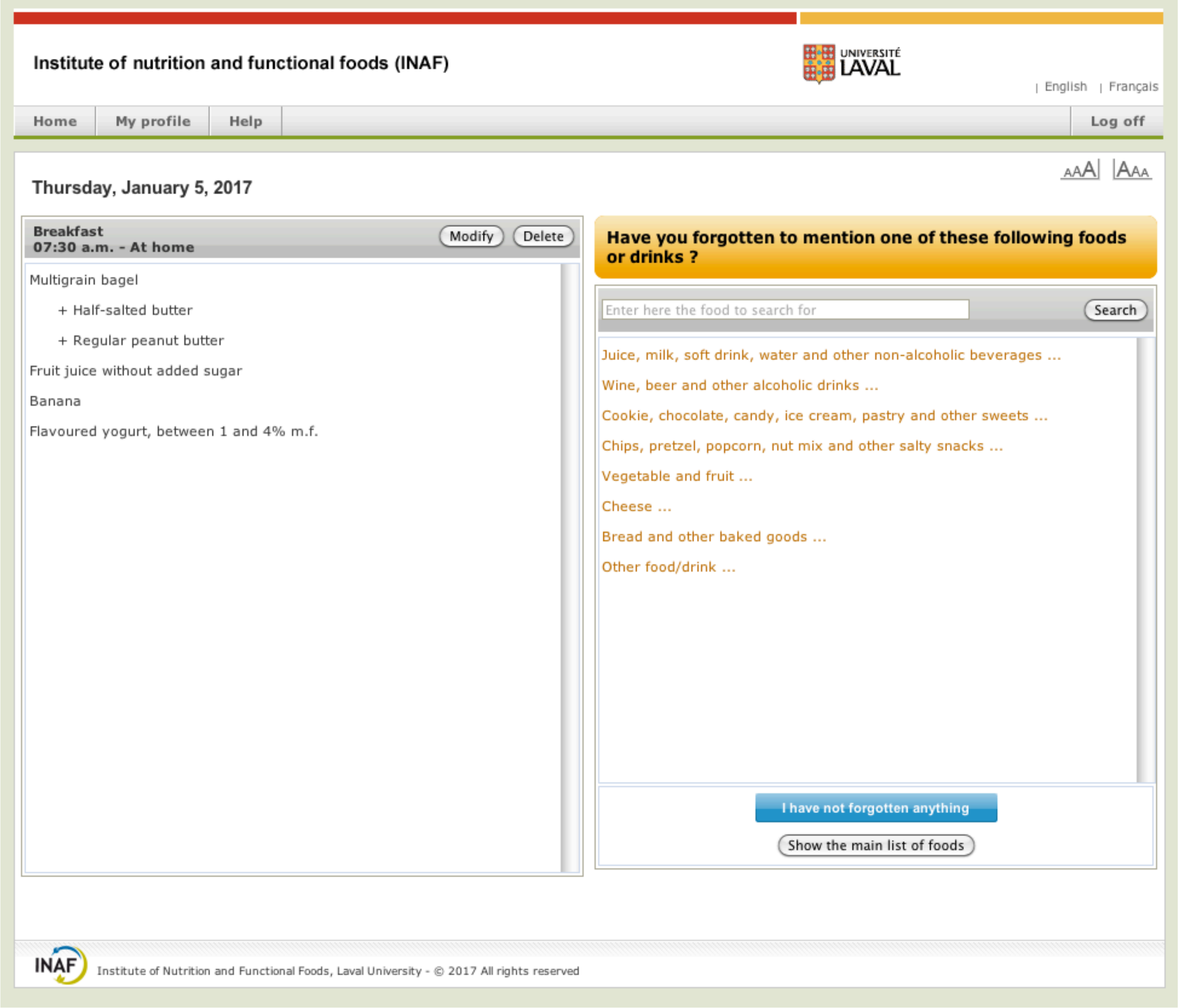

Figure 13: Verification of the summary of the day

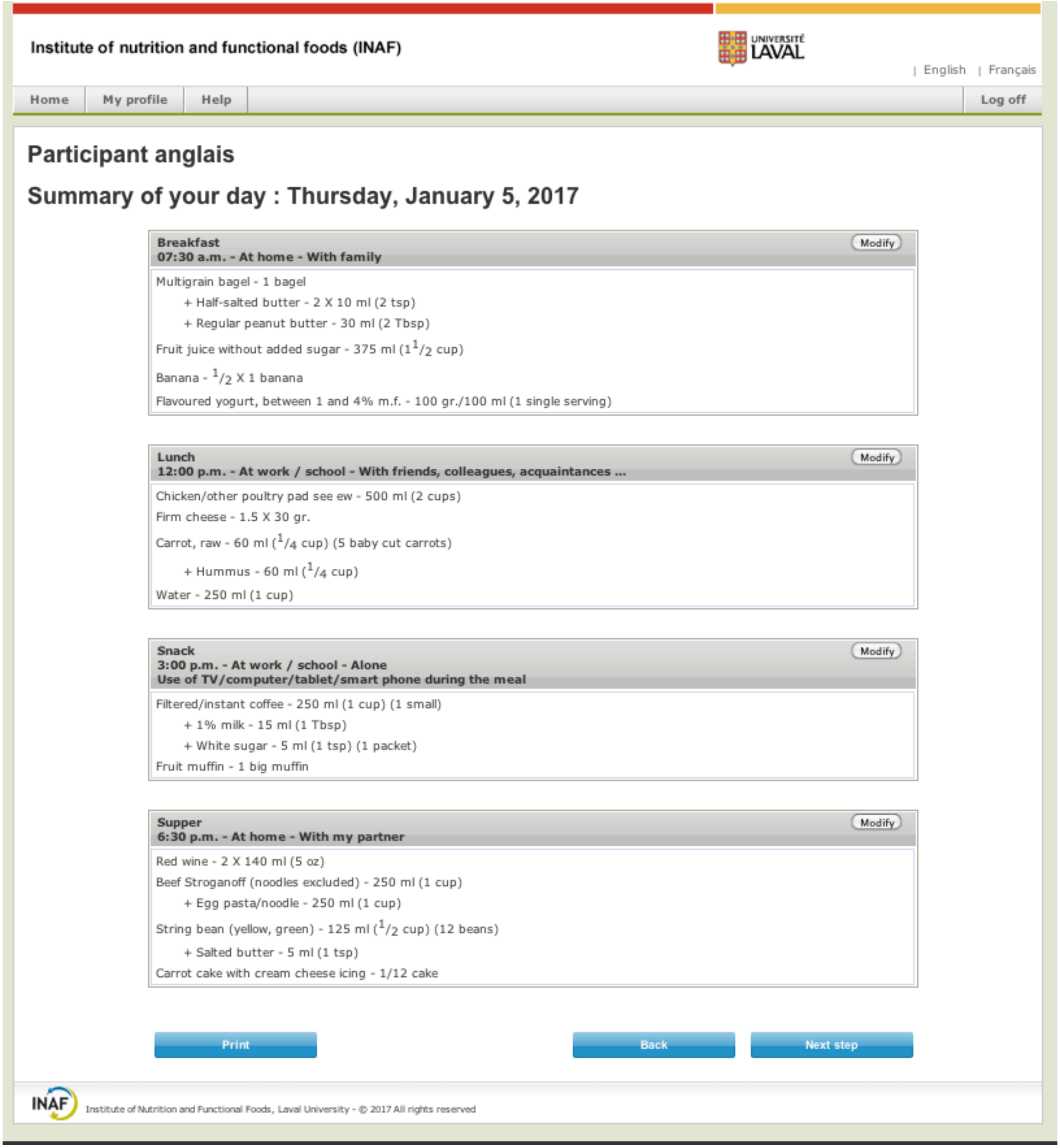

Figure 14: Complementary information

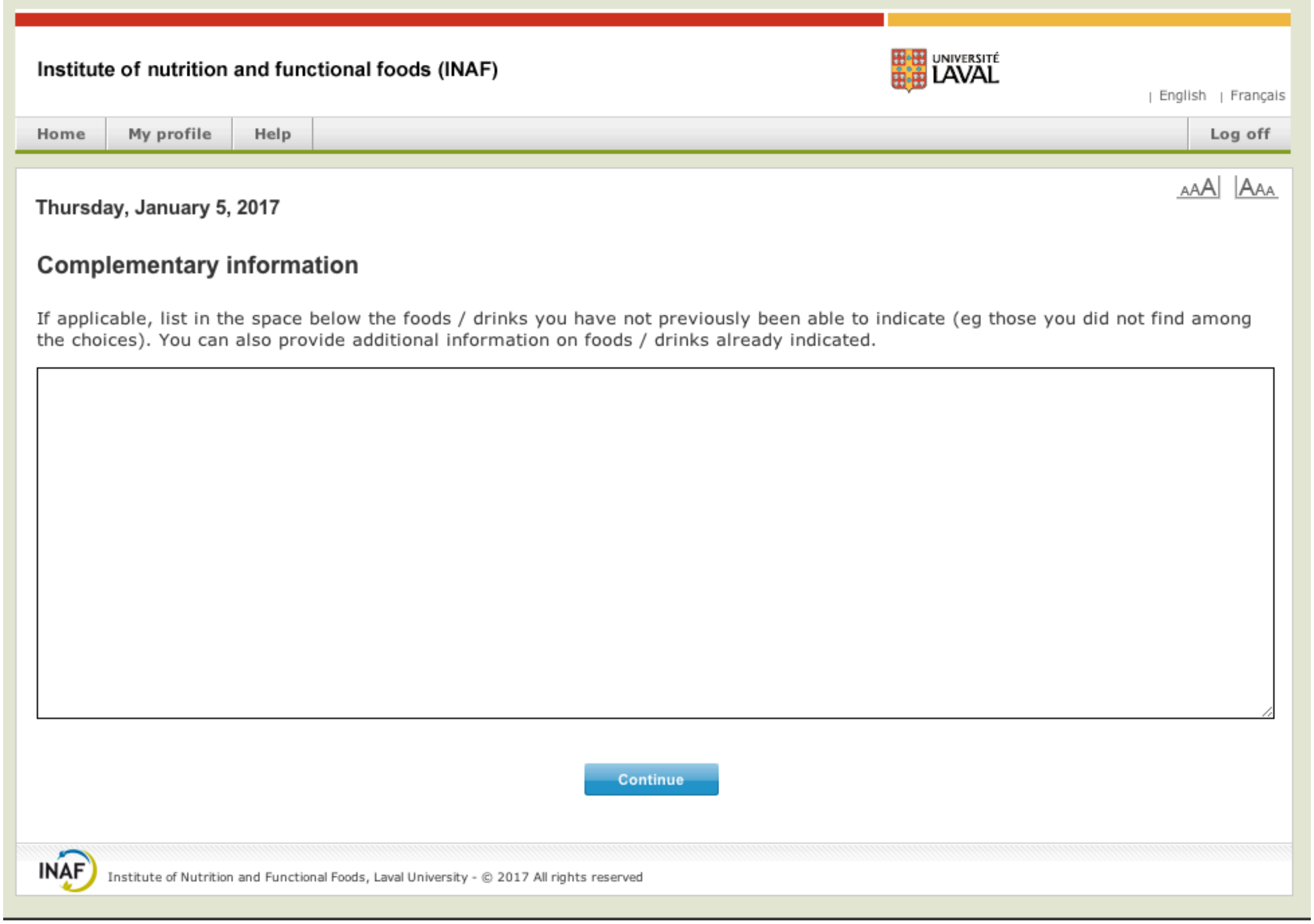

Figure 15: Representativeness of the day

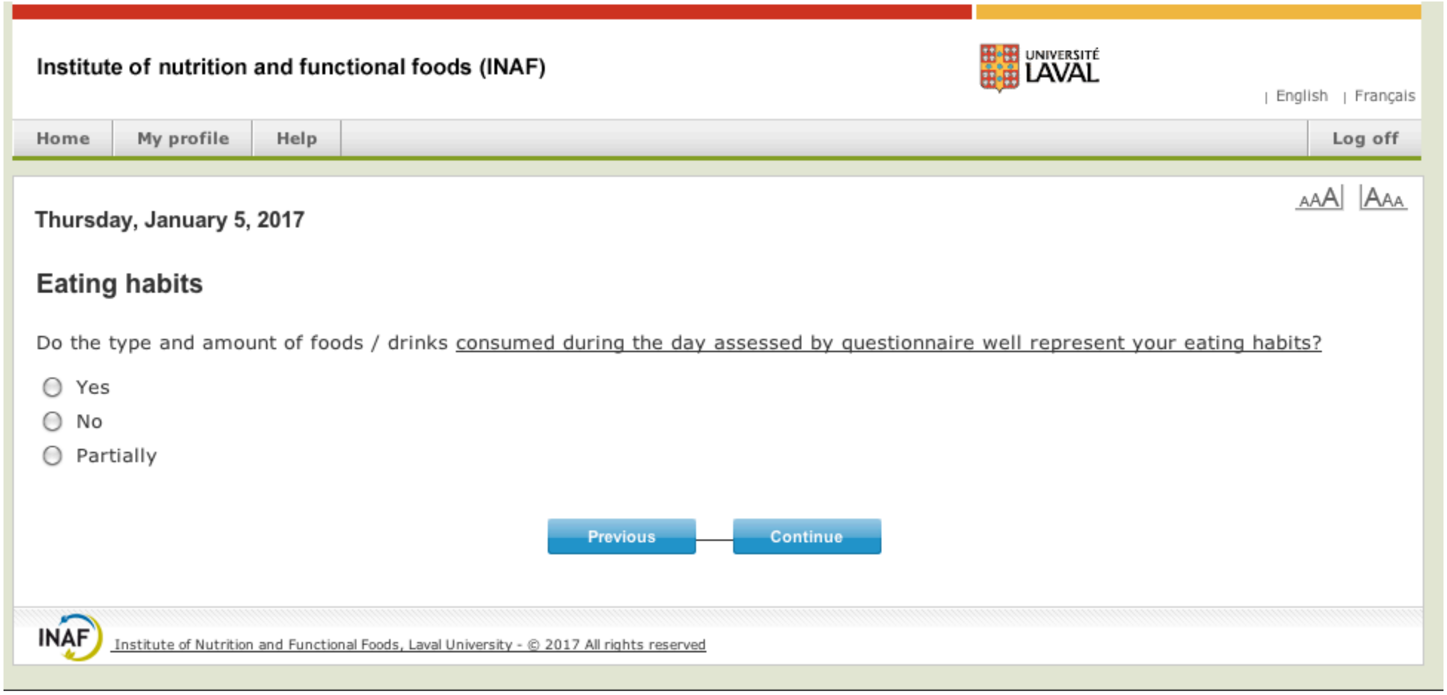

Figure 16: Age category

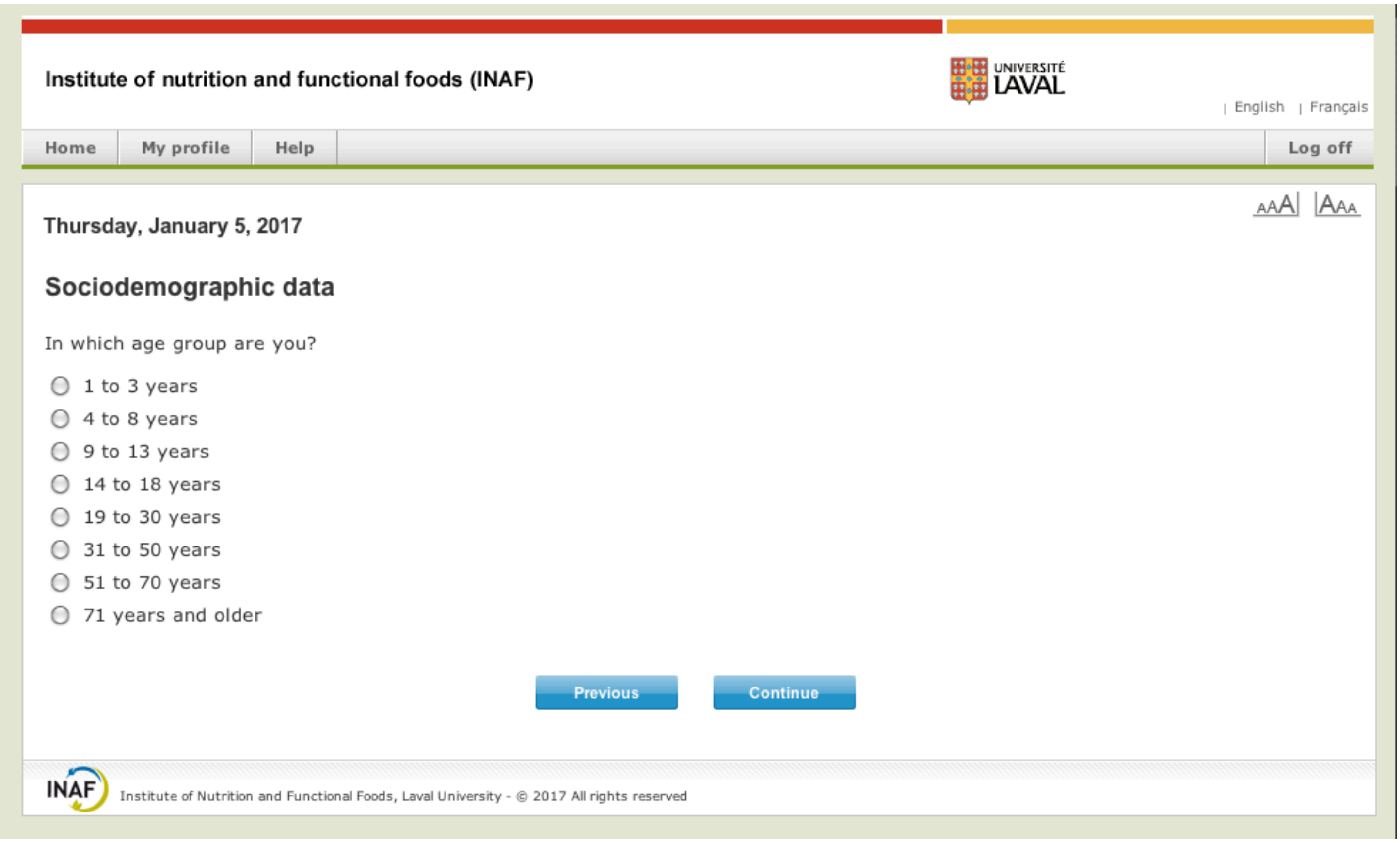

Figure 17: Use of supplements

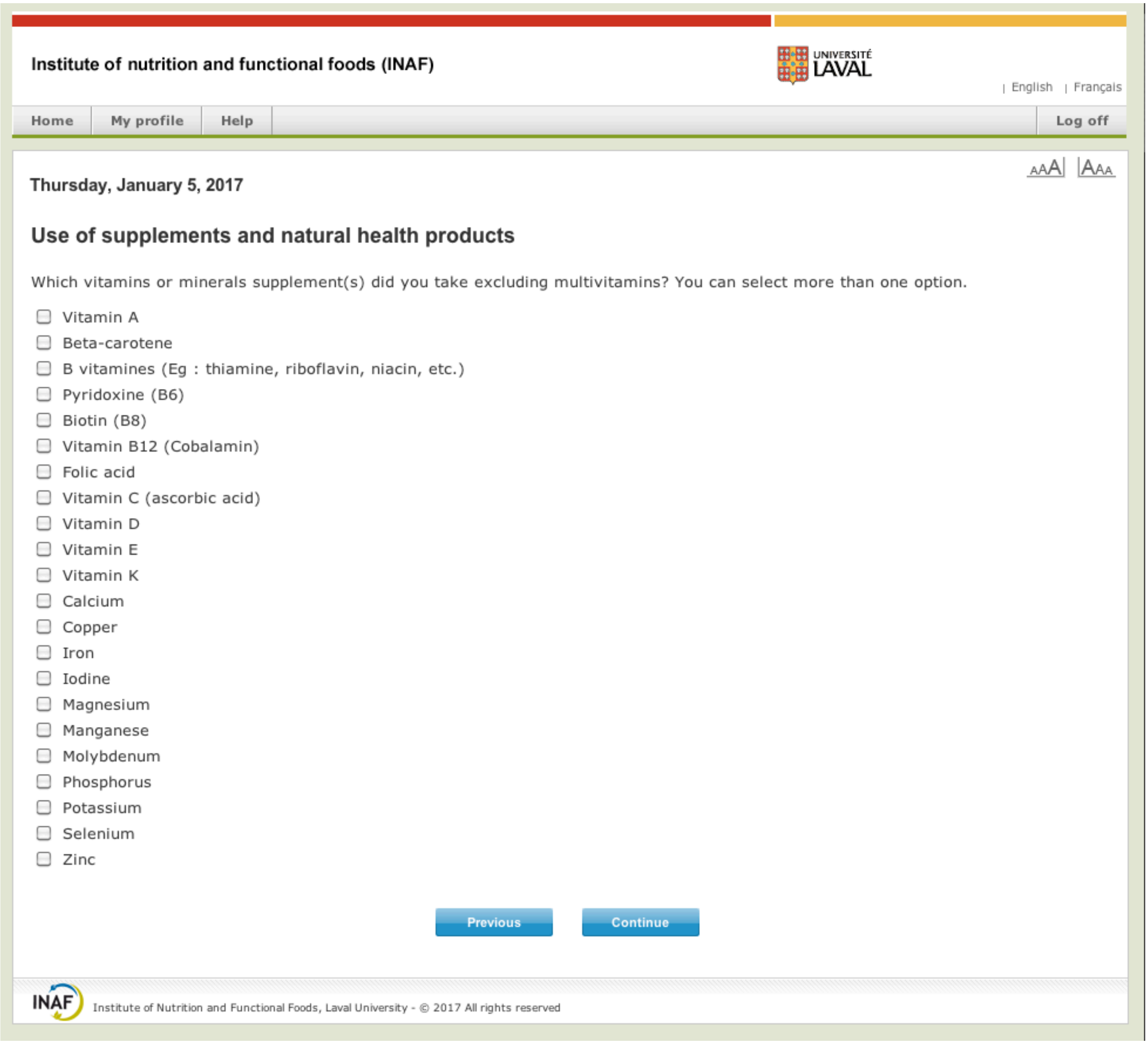

Institute of nutrition and functional foods (INAF)

UNIVERSITÉ LAVAL

| English | Français

Home | My profile | Help | Log off

AAA AAA

Thursday, January 5, 2017

Use of supplements and natural health products

Which vitamins or minerals supplement(s) did you take excluding multivitamins? You can select more than one option.

- ☐ Vitamin A
- ☐ Beta-carotene
- ☐ B vitamines (Eg : thiamine, riboflavin, niacin, etc.)
- ☐ Pyridoxine (B6)
- ☐ Biotin (B8)
- ☐ Vitamin B12 (Cobalamin)
- ☐ Folic acid
- ☐ Vitamin C (ascorbic acid)
- ☐ Vitamin D
- ☐ Vitamin E
- ☐ Vitamin K
- ☐ Calcium
- ☐ Copper
- ☐ Iron
- ☐ Iodine
- ☐ Magnesium
- ☐ Manganese
- ☐ Molybdenum
- ☐ Phosphorus
- ☐ Potassium
- ☐ Selenium
- ☐ Zinc

Previous | Continue

INAF Institute of Nutrition and Functional Foods, Laval University - © 2017 All rights reserved

Figure 18: Use of natural health products

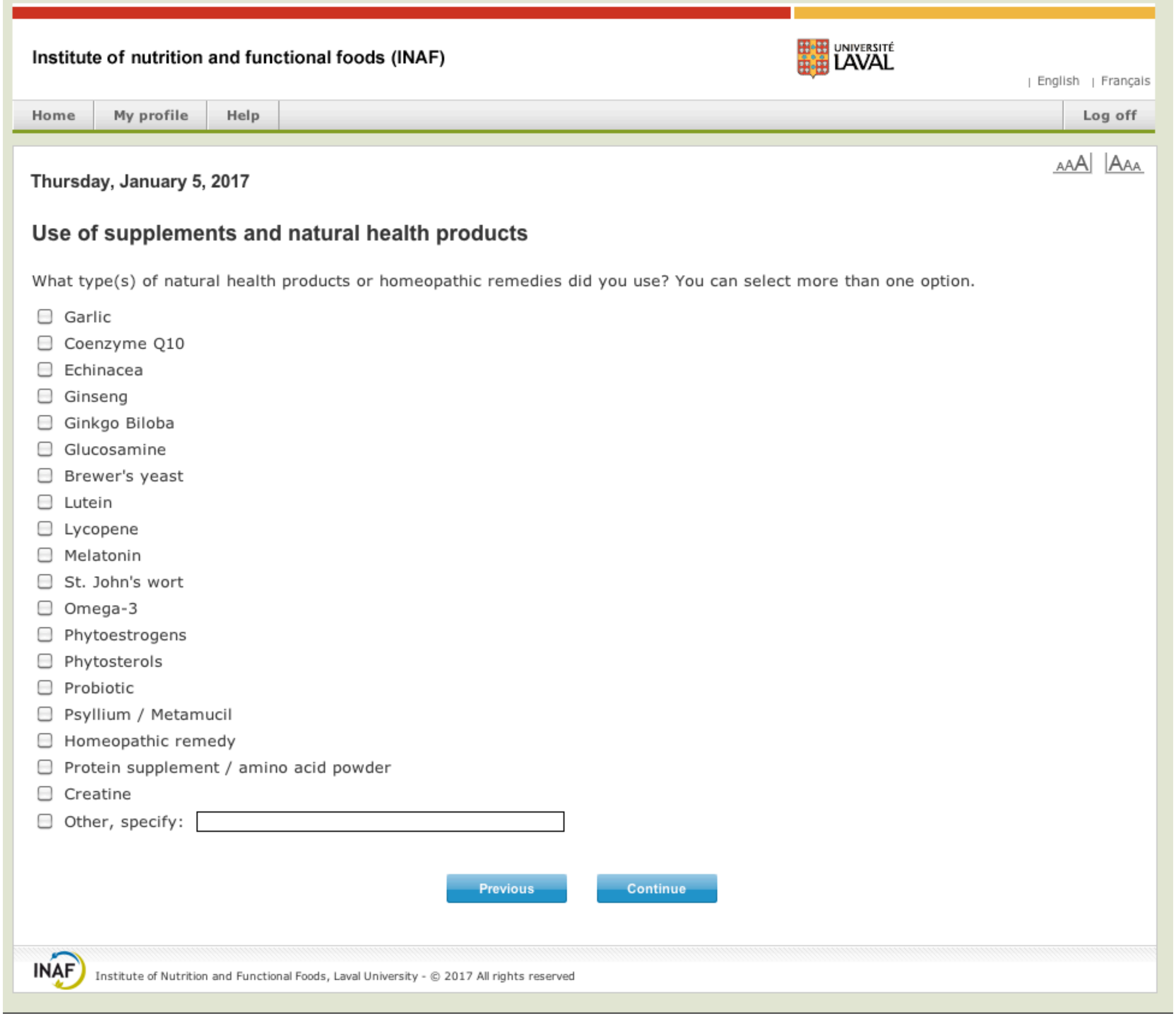

Institute of nutrition and functional foods (INAF)

UNIVERSITÉ LAVAL

| English | Français

Home | My profile | Help | Log off

Thursday, January 5, 2017

Use of supplements and natural health products

What type(s) of natural health products or homeopathic remedies did you use? You can select more than one option.

- ☐ Garlic
- ☐ Coenzyme Q10
- ☐ Echinacea
- ☐ Ginseng
- ☐ Ginkgo Biloba
- ☐ Glucosamine
- ☐ Brewer's yeast
- ☐ Lutein
- ☐ Lycopene
- ☐ Melatonin
- ☐ St. John's wort
- ☐ Omega-3
- ☐ Phytoestrogens
- ☐ Phytosterols
- ☐ Probiotic
- ☐ Psyllium / Metamucil
- ☐ Homeopathic remedy
- ☐ Protein supplement / amino acid powder
- ☐ Creatine
- ☐ Other, specify: ________

Previous | Continue

INAF Institute of Nutrition and Functional Foods, Laval University - © 2017 All rights reserved
